# Serum miRNA signature diagnoses and discriminates murine colitis subtypes and predicts ulcerative colitis in humans

**DOI:** 10.1038/s41598-017-02782-1

**Published:** 2017-05-31

**Authors:** Emilie Viennois, Yuan Zhao, Moon Kwon Han, Bo Xiao, Mingzhen Zhang, Meena Prasad, Lixin Wang, Didier Merlin

**Affiliations:** 1 Institute for Biomedical Sciences, Center for Diagnostics and Therapeutics, Georgia State University, Atlanta, GA 30303 USA; 2 Department of Gastroenterology, Zhongshan Hospital, Fudan University, Shanghai, China; 3 Institute for Clean Energy and Advanced Materials, Faculty for Materials and Energy, Southwest University, Chongqing, 400715 China; 4 Veterans Affairs Medical Center, Decatur, GA USA; 5 Emory University, Department of Medicine, Atlanta, GA USA

## Abstract

Inflammatory bowel disease (IBD) is difficult to diagnose due to nonspecific and variable symptoms, and lack of reliable diagnostic tests. Current methods are invasive, non-sensitive, non-predictive, and do not easily discriminate between its two main forms. Consequently, there remains a great need for reliable serum markers for IBD. Here, using a longitudinal study of various mouse models of colitis, we identified a serum miRNA signature that indicated the development of colitis and discriminated between inflammations of various origins (colitis from arthritis). Unlike the existing biomarkers, the newly identified signature also serves to distinguish individuals at risk, predict the type of inflammation, and evaluate the response to therapeutics. Moreover, the miRNA signature identified in mice predicted ulcerative colitis with 83.3% accuracy. In future, the signature identified herein could play a central role in monitoring inflammatory disorders and therapeutic responses in patients, thereby paving the way for personalized medicine.

## Introduction

In human, many disorders are difficult to diagnose due to presence of nonspecific and variable symptoms, and lack of reliable diagnostic tests. Some conditions such as inflammatory bowel disease (IBD) are difficult to diagnose because there is no real test to indicate their existence; rather, it is diagnosed indirectly by excluding other conditions.

IBD for which the two major clinical forms are ulcerative colitis (UC) and Crohn’s disease (CD), is a chronic inflammatory disorder that affects the gastrointestinal (GI) tract^1^. IBD is a substantial public health concern that leads to considerable human suffering, productivity losses, and high health costs. Furthermore, intestinal inflammation associated with IBD and other causes is thought to underlie a significant portion of colonic neoplasia, which is a leading cause of mortality in developed countries^2, 3^. With an increasing incidence of IBD in Europe and North America (36–40 new cases per 100,000 individuals per year)^4^, as well as in developing countries^5, 6^, its diagnosis and assessment remains an important challenge.

An inquiry of patient medical history, description of symptoms (abdominal pain, cramping, diarrhea, rectal bleeding, and extreme fatigue), physical examination and a combination of tests that includes blood tests and endoscopic procedure is the conventional diagnosis process, with colonoscopy usually prescribed to assess the endoscopic appearance of the colon. However, this technique is not ideal for monitoring disease activity: it is expensive, burdensome for patients, requires sedation, and carries a small (0.05%) but relevant risk of serious complications, including perforation and death^7^. Blood tests and marker analysis may be used to complement the endoscopic procedures^8^. For example, C-reactive protein (CRP) is strongly correlated with CD, but less so with UC^9, 10^. Various serologic tests have been examined in an effort to improve the diagnosis of IBD and find ways to unequivocally distinguish between CD and UC. Such tests may include the determination of perinuclear anti-neutrophil cytoplasmic antibodies (pANCAs) and anti-Saccharomyces cerevisiae antibodies (ASCAs)^9, 11^. However, none of these markers has proven ideal for the assessment of IBD. Calprotectin and lipocalin-2 (Lcn-2) are two other recently reported sensitive and broadly dynamic fecal markers of inflammation^12, 13^. However, a clinical use of these biomarkers has been limited by problems with non-specificity (they are increased under any inflammatory condition, including cancer, liver injury, and gut damage^14, 15^), the absence of predictive aspects, and difficulties in making a differential diagnosis between UC and CD. Therefore, as for many other chronic inflammatory conditions, there is a need for biomarkers that are both sensitive and non-invasive that can facilitate the diagnosis, ideally before the onset of clinical symptoms. In addition, an ideal laboratory biomarker should be disease-specific, able to identify individuals at risk for the disease, able to quantify disease activity, able to monitor the therapeutic response, and prognostically valuable for assessing disease relapse or recurrence^8^. Since no single biomarker introduced to date has possessed all of the desired qualities, researchers have begun seeking to unravel a relevant biomarker signature^16, 17^.

Circulating microRNAs (miRNAs) have recently emerged as promising non-invasive biomarkers for IBD. miRNAs are small noncoding RNAs (18–25 nucleotides) that play important roles in regulating various biological processes *via* the post-transcriptional gene expression changes induced by their binding to the 3′-untranslated regions of target mRNAs^18^. miRNAs are present in extracellular human body fluids (*e.g*., serum, plasma, saliva, and urine) in a highly stable form, making them a promising candidate for use as blood biomarkers for IBD and various other diseases^19^. miRNAs are frequently dysregulated in pathological conditions, and reports have shown that they have a great potential as markers for various diseases, mostly cancers^20–22^.

Dysregulation of miRNA expression in colonic mucosa samples from IBD patients was first reported in 2008^23^. Since then, other studies have identified differential expressions of miRNA panels in colonic tissues/biopsies, and have sought to use these panels to distinguish active *vs*. quiescent UC or CD^24–26^, or indeterminate colitis from CD^27^. However, the analysis of miRNA profiles in colonic tissues requires that biopsies be collected by colonoscopy, making this an invasive diagnostic method. Also, none of the studies identified a miRNA panel sensitive enough to effectively distinguish active CD from UC, predict disease susceptibility, or assess the therapeutic response.

The present study was designed to assess longitudinal alterations of serum miRNAs during the development of intestinal inflammation in mouse models, and determine whether such modifications may accurately be used to diagnose and follow the development of colitis, as well as the response to therapy. Together, our results develop a general concept of miRNA signature for a better diagnosis of human disorders by describing a novel and unique miRNA profile for intestinal inflammation.

## Results

### A panel of circulating miRNAs is associated with colitis progression in IL10^−/−^ mice

IL-10 is a well-known suppressor of TH1 cells and macrophage effector functions. *In vitro* studies have shown that IL-10 inhibits the production of IL-12 and Tumor Necrosis Factor-alpha (TNF-α), and T cell proliferation, and may also promote the formation of antigen-specific regulatory T cells^28, 29^. IL-10^−/−^ mice spontaneously develop chronic enterocolitis with massive infiltration of lymphocytes, activated macrophages, and neutrophils^30^. To confirm the usefulness of the IL10^−/−^ model as a tool for investigating miRNA expression profiles during intestinal inflammation, we characterized the development of colitis in these mice. Female IL10^−/−^ mice were monitored for colitis development for 98 days, from weaning at day 30 (D30) to 98 days post-weaning (D98). Histological features were assessed by hematoxylin-eosin staining, and the degree of intestinal inflammation was measured using fecal Lcn-2, which was previously described as being a sensitive and cost-effective fecal marker that is correlated with the severity of intestinal inflammation^13, 31^ (Fig. S1).

**Figure 1:**
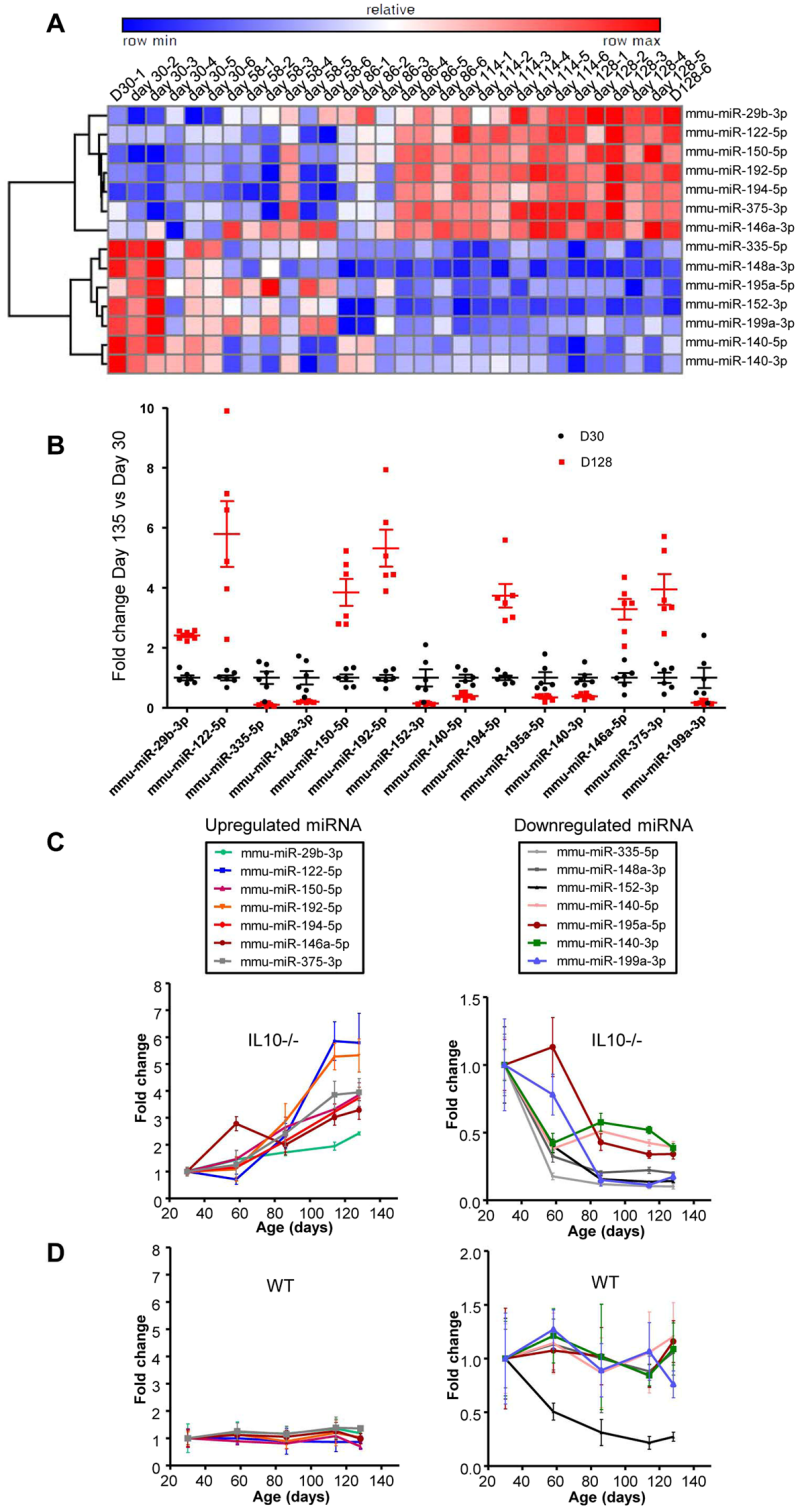
Serum miRNA profiles in a discovery cohort of IL10^−/−^ mice. Sera were collected from IL10^−/−^ mice at age 30, 58, 86, 114 and 128-days and subjected to miRNA profiling. (**A**) Hierarchical clustering showing the relative expression levels of the 14 deregulated miRNAs in each sample. (**B**) Expression profiles of miRNAs in colitic mice (D128) compared to the same mice before they develop colitis (D30). The average expression of each miRNA in non-colitic mice was set to 1. (**C**) Age-dependent representation of the expression profiles of the 14 deregulated miRNAs in the sera of IL10^−/−^ mice. Seven were increased (left panel), and seven were decreased (right panel). (**D**) The expression levels of these differentially expressed miRNAs were analyzed in sera from WT mice. Data are given as the means +/− S.E.M. (n = 6).

Hematoxylin-eosin staining of the colon revealed that 135-day-old (D135) IL10^−/−^ mice exhibited histological signs of chronic inflammation, characterized by immune cell infiltration and epithelial erosion (Fig. S1A). The levels of Lcn-2 were 6- and 130-fold higher in the feces of IL10^−/−^ mice *versus* Wild-type (WT) mice on days 40 and 127, respectively (Fig. S1B). This increase, which reflected the development of colitis, was found to be time-dependent, with the Lcn-2 concentration on day 127 being 30-fold higher than that on day 40 (5139 ng/g *versus* 170 ng/g of feces, respectively) (Fig. S1B). These results confirmed that IL10^−/−^ mice developed colitis in a time-dependent manner. Thus, we used IL10^−/−^ mice to address whether miRNA levels in serum could differentiate colitic from non-colitic individuals. To identify candidate serum miRNA biomarkers, we first performed miRNA profiling *via* high-throughput qPCR analysis of the sera of the same IL10^−/−^ mice aged 30 (D30), 58 (D58), 86 (D86), 114 (D114) and 128 (D128) days (Fig. S2). The number of miRNAs detected per sample (count) fluctuated from 111 to 129, and the average amplification threshold (Cp) ranged from 26.2 to 30.7; these findings were comparable between serum samples, suggesting that the samples were of similar quality and were processed reproducibly (Fig. S3A). Analysis of the technical internal controls, which consisted of the RNA spike-in, UniSp6, and the DNA spike-in, UniSp3, indicated that both reverse transcription and qPCR were successful (Fig. S3B). As it is known to potentially impact miRNA profiling, we monitored hemolysis in our samples using a sensitive method based on the calculation of ratio of miR-451a (enriched in erythrocytes) to miR-23a-3p (relatively stable in plasma and serum and not affected by hemolysis)^32^. Delta (miR-23a-3p - miR- 451a) were measured and possible erythrocyte microRNA contaminations were indicated by Delta Cq ≥ 5 (Orange), and Delta Cq ≥ 7–8 indicating a high hemolysis (red)^33^. Importantly, none of the used samples were having a high hemolysis level (Fig. S3C). Three samples had a ratio ≥ 5 showing a moderate risk of hemolysis. Of 138 assays, 104 miRNAs were detected in all of the samples. When we compared the five age groups using one-way ANOVA and a cutoff of Bonferroni *p-*value < 4.42E-04, we identified 33 miRNAs that were differentially expressed (Table S1). These miRNAs fulfilled the Bonferroni correction, which is a safeguard against false positives in these types of data set. The top 14 most significantly altered miRNAs, namely mmu-miR-29b-3p, -122-5p, -150-5p, -192-5p, -194-5p, -375-3p, -146a-3p, -335-5p, -148a-3p, -195a-5p, -152-3p, -199a-3p, -140-5p and -140-3p, are presented in the heat map shown in Fig. 1A, and were used for subsequent analysis. Seven were up-regulated in IL10^−/−^ colitic mice, and seven were down-regulated in these mice. In an effort to define a miRNA pattern that could be used to distinguish between colitic and non-colitic mice, we adjusted the average expression levels of the 14 miRNAs with respect to the non-colitic samples (D30) to 1, and represented the variations observed at D128 (colitic samples) as fold changes. Figure 1B shows the expression pattern of these miRNAs in colitic mice (D128 - red squares) compared to the same mice before they developed colitis (D30 - black points) highlighting the appearance of a “colitic-type” signature. Moreover, the profiles of these 14 miRNAs appeared to reflect the degree of inflammation and the evolution of the disease, since they each increased or decreased in progressive and time-dependent manners that resembled the development of intestinal inflammation in the IL10^−/−^ mice (Fig. 1C). In order to demonstrate that the alteration of the miRNAs in IL10^−/−^ was due to the development of colitis rather than an age effect, we analyzed the expression levels of the candidate miRNAs in the sera of WT mice at the same time points (Fig. 1D). Their expressions were not altered in WT mice, with the exception of miR-152, which was consistently lower than that in non-colitic IL10^−/−^ mice. Based on this, miRNA-152 was excluded from the signature. Thus, we tested a panel of 13 miRNAs as potential markers of intestinal inflammation.

### Validation of miRNA signature of colitis

**Figure 2:**
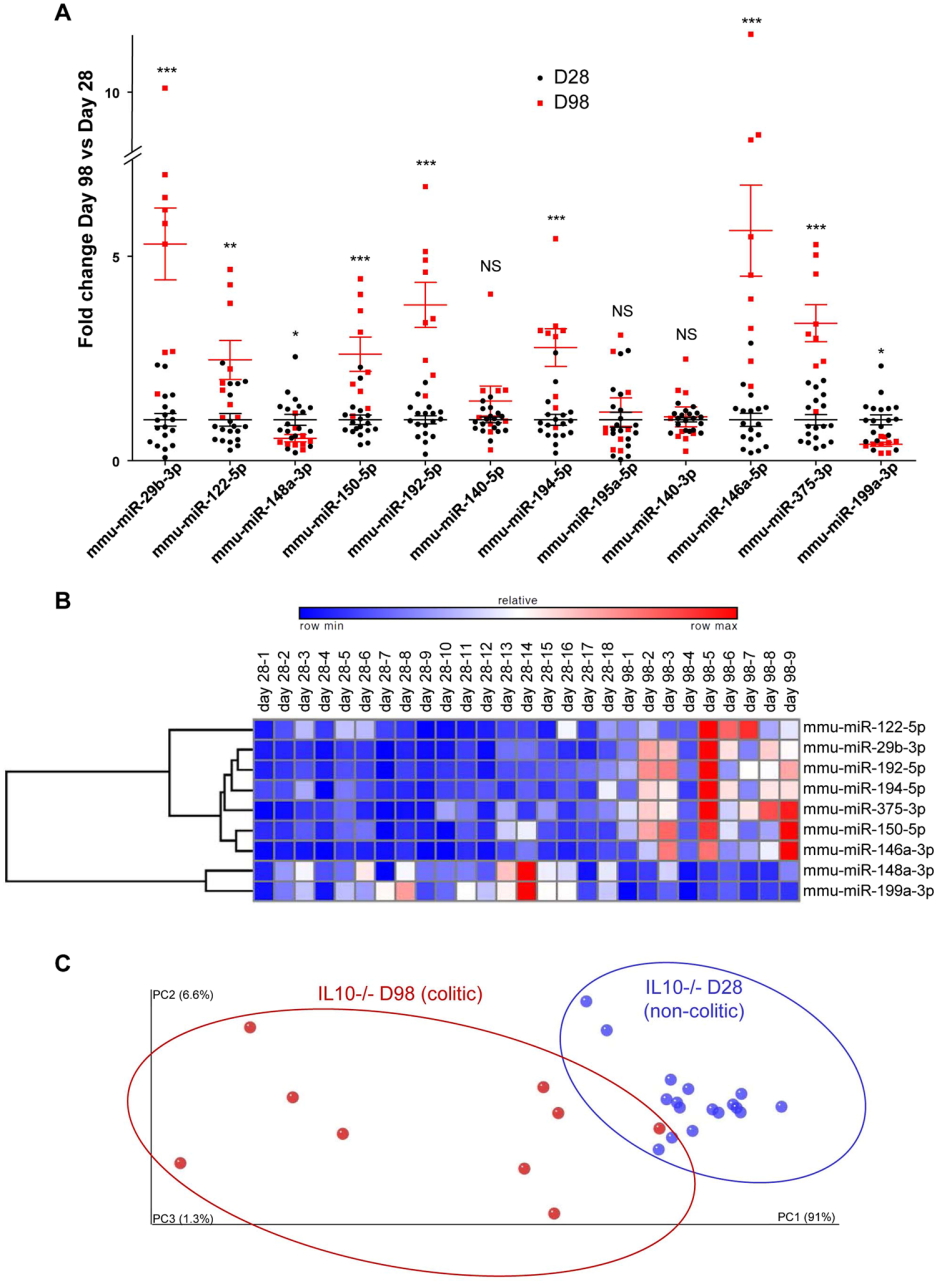
Validation of a new miRNA signature for the assessment of colitis in a validation cohort of IL10^−/−^ mice. The expression levels of 13 deregulated miRNAs were validated by qPCR using a second and independent set of IL10^−/−^ mice. (**A**) Fold changes of the 12 expressed miRNAs in this new set of IL10^−/−^ colitic mice (D98) compared to IL10^−/−^ non-colitic mice (D28). The expression level of each miRNA was analyzed and standardized to 1 in non-colitic mice. (**B**) Hierarchical clustering showing the relative expression levels of the nine miRNAs in each sample. (**C**) Principal coordinate analysis (PCoA) plot of the euclidean distance matrix for miRNA expression in sera was performed using QIIME software and plotted using Emperor Software. Percentages on the axes indicate the percentage of variance explained by each component. The populations of the nine selected miRNAs in 28 day-old (blue) or 98 day-old (red) IL10^−/−^ mice were clustered using normed PCoA. Permanova test, *p* = 0.001. Data are presented as the means +/− S.E.M. (n = 9–18). Significance was determined using unpaired t-test (*p < 0.05; **p < 0.01; ***p < 0.001; NS, not significantly different) compared to the results obtained from the corresponding D28 IL10^−/−^ mice.

To validate our miRNA signature, we used it to analyze an independent set of IL10^−/−^ mice. qPCR was used to analyze the serum expression levels of the 13 deregulated miRNAs (Table S2, Fig. 2A) on D98 (after the establishment of colitis) compared to D28 (before the development of colitis). Of the 13 miRNAs, nine showed the same differential expressions in sera of colitic IL10^−/−^ *versus* non-colitic IL10^−/−^ mice in the second set of animals. The remaining four miRNAs were either not detected in this new set of mice (miR-335-5p) or did not show a significant change of the expression levels (miR-140-5p, miR-195a-5p and miR-140-3p) (Fig. 2A). These differences probably reflected a larger number of replicates used for our validation analysis (n = 9 to 18 compared to n = 6 in the high-throughput analysis). The four miRNAs whose differential expressions were not confirmed in this experiment were excluded from the panel.

Our validated colitis signature was therefore composed of nine miRNAs: the up-regulated miRNAs, miR-29b-3p, -122-5p, -192-5p, -194-5p, -375-3p, -150-5p, and -146a-3p, and the down-regulated miRNAs, miR-148a-3p and -199a-3p (Fig. 2B). This nine-miRNA signature was found to be sufficient to segregate colitic *versus* non-colitic IL10^−/−^ mice, as revealed by our principal coordinate analysis (PCoA)-based assessment of the variations between individual mouse replicates, which showed that there was a significant difference (Pemanova test, *p* = 0.001) in the patterns of clustering between D28 (blue) and D98 (red) (Fig. 2C). Thus, using IL10^−/−^ mice, we successfully identified a serum miRNA signature that could be used to identify mice with intestinal inflammation.

### miRNA signature specifically identifies intestinal inflammation

**Figure 3:**
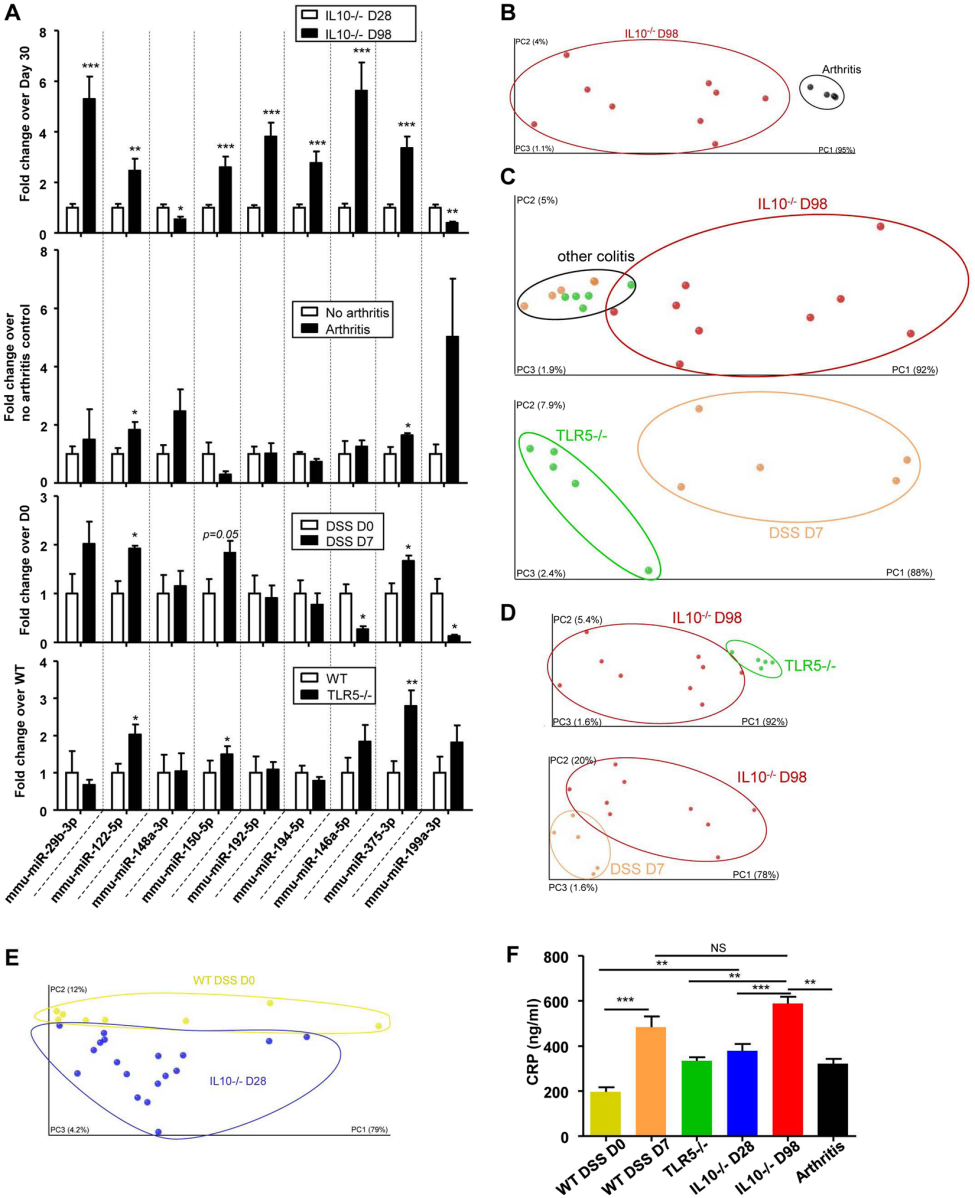
New miRNA signature specificity for intestinal inflammation and discrimination between different subtypes of colitis. Sera representing three different mouse models of colitis (IL10^−/−^, TLR5^−/−^ and DSS-induced colitis) and arthritic mice were collected. (**A**) Fold changes of the nine signature miRNAs in IL10^−/−^ (D98), arthritic (day 12) mice, 3% DSS-induced-colitic and TLR5^−/−^, compared to the relevant controls [28 day-old IL10^−/−^ mice, Balb/C mice before the establishment of arthritis (day 2), water-control C57BL/6 WT mice and C57BL/6 WT mice]. (**B**–**E**) PCoA of the euclidean distance matrix of the 9 miRNA expression in sera of various mouse models was performed using QIIME software and plotted using Emperor Software. Percentages on the axes indicate the percentage of variance explained by each component. (**B**) IL10^−/−^ mice at D98 (red) compared to sera of arthritic (black) mice; (**C**), IL10^−/−^ colitic mice (day 98, red), TLR5^−/−^ mice (green) and DSS-induced colitic mice (orange); (**D**), IL10^−/−^ colitic mice on day 28 (blue) and D98 (red) compared to TLR5^−/−^ and DSS-induced colitic mice; and (**E**) IL10^−/−^ mice at D28 (blue) compared to WT water-treated control mice (DSS D0, yellow). (**F**) CRP levels were measured in the sera of WT, D7 DSS, TLR5^−/−^, D28 IL10^−/−^, D98 IL10^−/−^ and arthritic mice. Data are presented as the means +/− S.E.M. (n = 5–18). Significance was determined using unpaired t-test (A) or using One-way ANOVA followed by a Bonferroni post-test (**F**) (*p < 0.05; **p < 0.01; ***p < 0.001; and NS, not significantly different) compared to the corresponding non-inflamed controls.

We next questioned whether the identified miRNA signature was specific to intestinal inflammation, or if it might be a general marker of inflammation. We used collagen antibody-induced arthritis (CAIA) as a model of extra-intestinal inflammation. Lcn-2 was increased in the serum of arthritic mice at day 12, and the clinical score was higher on day 12 compared to the pre-treatment time point (day 2) (Fig. S4A). Expressional analysis of the nine-miRNA signature in sera of CAIA mice revealed that two of the miRNAs (miR-122-5p and miR-375) were increased in arthritic mice compared to non-arthritic control mice (day 2) as it was observed in the IL10^−/−^ mice (Fig. 3A). To validate the specificity of our signature for intestinal inflammation, we used PCoA to show that our signature could distinguish IL10^−/−^ mice at D98 (colitic) from arthritic mice (black) (Fig. 3B).

Next, to investigate whether the identified miRNA signature could discriminate intestinal inflammation of different origins, we tested two other mouse models of colitis: dextran sodium sulfate (DSS)-induced acute colitis^13, 34^ and the TLR5^−/−^ mouse line, a previously described model of intestinal inflammation^35^. Mice treated for 7 days with 3% DSS developed acute colitis, as evidenced by their fecal Lcn-2 levels (Fig. S4B). Consistent with the previous report^35^, only a subset of the TLR5^−/−^ mice developed spontaneous chronic colitis. Mice with serum Lcn-2 levels of 150 ng/ml and above were classified as colitic (Fig. S4C) and used for analysis as reported before. One TLR5^−/−^ sample showed a modest risk of hemolysis (Fig. S4D). Analysis of the expression levels of the nine-miRNA panel revealed that three miRNAs (miR-122-5p, miR-150-5p, and miR-375) were similarly altered (all up-regulated) in mice with DSS-induced and TLR5^−/−^ colitis compared to the corresponding healthy controls (Fig. 3A). Of the remaining miRNAs, miR-199a-3p was down-regulated in DSS induced colitis (and the IL10^−/−^ model), but it was unaltered in the TLR5^−/−^ model. The other miRNAs were either unaltered or altered in the opposite direction from that seen in the IL10^−/−^ model, suggesting they were not generalizable (Fig. 3A). The expression of miR-150, which was increased in the three models of intestinal inflammation (IL10^−/−^, DSS, and TLR5^−/−^), was unaltered in the arthritis model, as were the remaining miRNAs of the signature (Fig. 3A).

Thus, among the nine miRNAs of the identified signature, two were deregulated in both intestinal inflammation and arthritis (miR-122-5p and miR-375), one was specifically deregulated in all three intestinal inflammation models (miR-150-5p), and the others appeared to be specific to the IL10^−/−^ mouse model. PCoA yielded a different pattern of clustering between IL10^−/−^ colitic mice (red, D98) and the other models of colitis [orange, DSS treated mice on day 7 (D7); and green, TLR5^−/−^] (Fig. 3C). A closer look on other colitis models showed that the signature can also be used to distinguish across the various models: between TLR5^−/−^ (green) and DSS D7 (orange) mice (Fig. 3C), between IL10^−/−^ D98 mice (after colitis, red) and TLR5^−/−^ mice (green) (Fig. 3D, upper panel) and between DSS D7 (orange) and IL10^−/−^ D98 mice (Fig. 3D, lower panel). On the other hand, CRP and Lcn-2 were not always altered between these different colitis models (Figs 3F and S5). These data suggested that our nine-miRNA signature could be used to discriminate among the different IBD models in a more sensitive manner than the current markers of inflammation.

When miRNA profiling was performed on young WT and IL10^−/−^ mice without any histological sign of intestinal inflammation, PCoA showed a difference in the patterns of clustering between non-DSS-treated WT mice (yellow) and non-colitic IL10^−/−^ mice (D28 in blue) (Fig. 3E). This may reflect the presence of a low-grade inflammation, as observed by increased CRP level (but not Lcn-2 level) in non-colitic IL10^−/−^ mice (D28) compared to WT mice, even in the absence of any histological features of inflammation (Figs 3F and S5). This latter finding shows that disease pattern in non-colitic IL10^−/−^ mice (*i.e*., at an age before the development of colitis) behave differently from non-genetically susceptible WT mice. Moreover, our results suggest that genetic predisposition could be reflected in a specific pre-existing miRNA signature as it is the case for some (but not all) existing biomarkers (such as CRP), and which could potentially serve as a preventive tool for early diagnosis.

**Figure 4:**
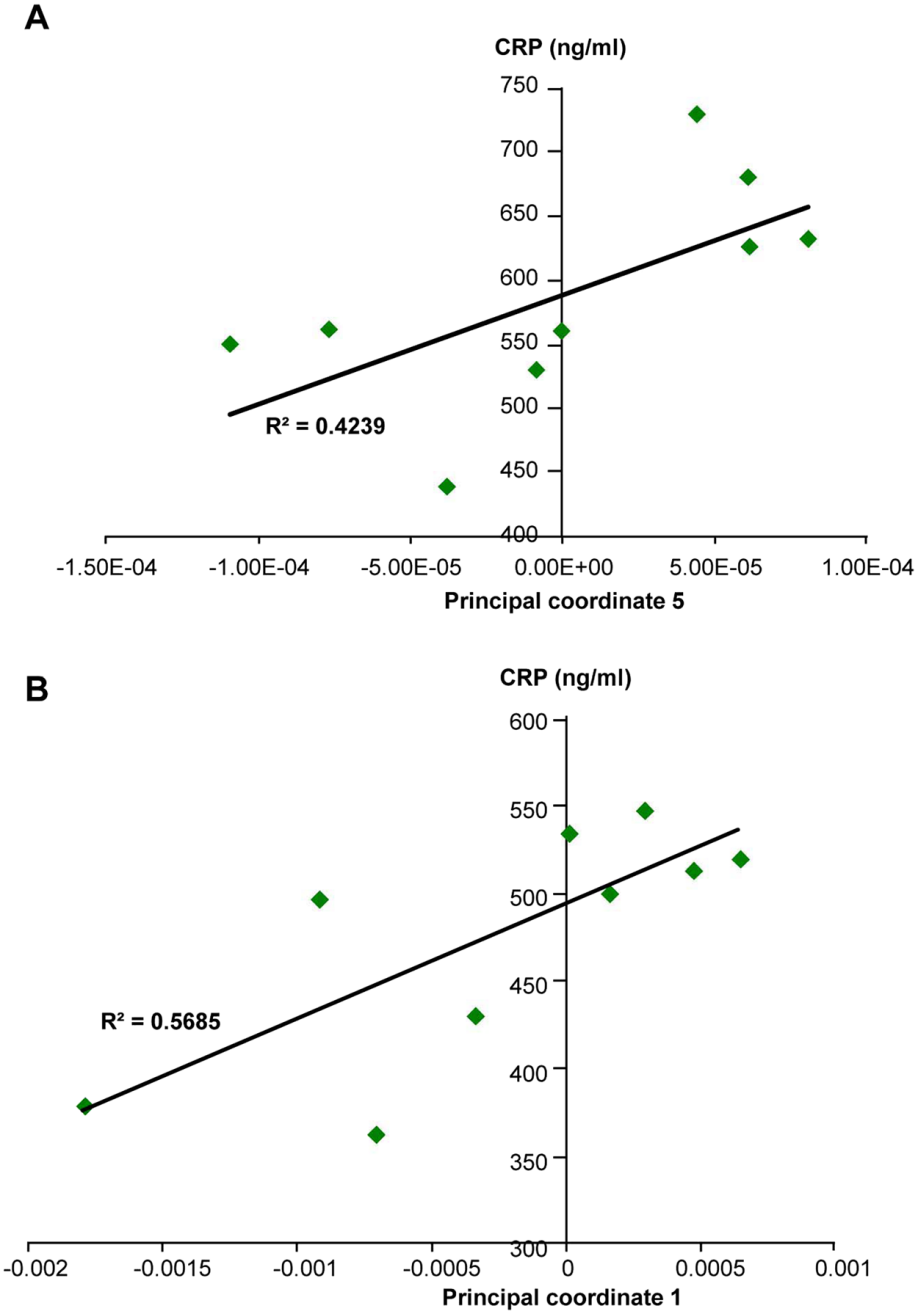
Correlation analysis between principal coordinates from the new miRNA signature and intestinal inflammation marker CRP. (**A**) Correlation analysis between principal coordinate 5 from the miRNA signature in IL10^−/−^ D98 mice and intestinal inflammation marker CRP. (**B**) Correlation analysis between principal coordinate 1 from the miRNA signature in anti-TNFα treated IL10^−/−^ D98 mice and intestinal inflammation marker CRP (n = 9). Significance was determined using an unpaired t-test (*p < 0.05; and **p < 0.01) compared to mice with the colitic-type signature.

In order to assess if our miRNA signature can offer some quantitative aspect, we further identified that some principal coordinate from the miRNA signature correlated with CRP values (R^2^ = 0.4239, P = 0.0574, Fig. 4A), highlighting that the identified miRNA signature can offer quantitative aspects, that can be used on an index scale, such as the CDAI (Crohn’s Disease Activity Index^36^).

Together, those results demonstrate that the nine-miRNA signature described herein has a potent discriminatory ability and a good specificity for the presence of different types of intestinal inflammation, and thus may have the potential to distinguish between the subtypes of IBD in humans.

To ensure that the differences in the miRNA expression profiles observed between colitic and non-colitic mice were not due to any confounding factors, we analyzed the miRNA panel in sera from mice housed in different cages or with different genetic backgrounds (Fig. S6A and B). PCoA did not show any clear clustering between the mice housed in different cages (Fig. S6A). Similarly, WT mice of different genetic backgrounds (BALB/C or C57/BL6) did not show any difference in the serum miRNA profile (Fig. S6B). These results confirmed that the newly identified miRNA signature was not influenced by any confounding factors.

### The miRNA signature indicates the response to therapeutics

**Figure 5:**
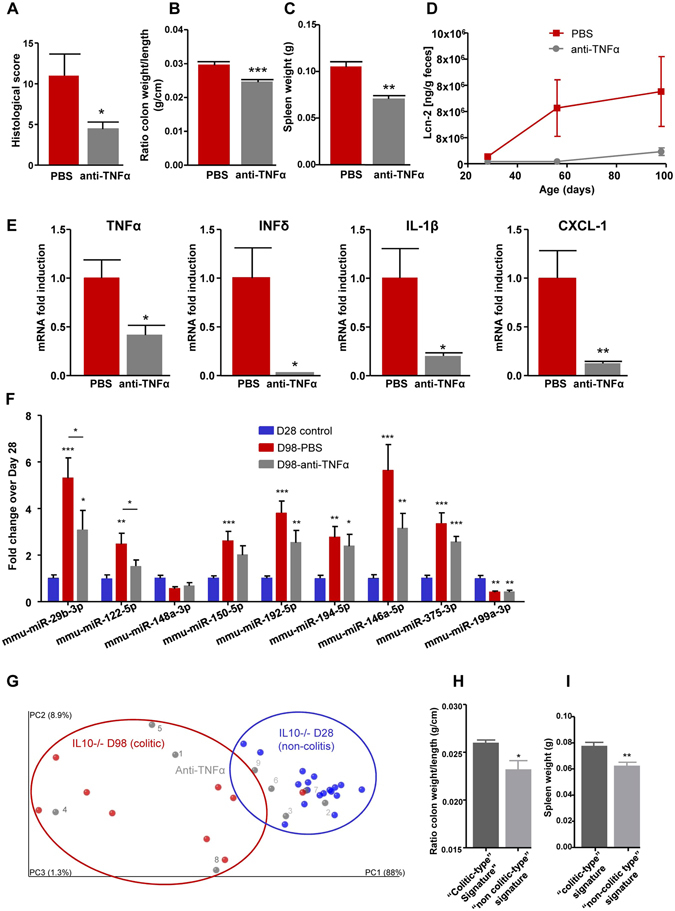
The miRNA signature in the evaluation of therapeutic response. IL10^−/−^ mice were treated by IP injection of anti-TNFα antibodies at a dose of 10 mg/kg two times per week for 10 weeks. (**A**) Histological scores were measured from H&E-stained colons (Fig. S7A). (**B**–**D**), spleen weight (**B**), ratio of colon weight/colon length (**C**), and fecal Lcn-2 (**D**) were measured. (**E**) Total RNAs were extracted from colons, and the expression levels of TNFα, IFN-δ, IL-1β and KC were quantified by qPCR and normalized with respect to 36B4. (**F**) The fold changes of the nine signature miRNAs were quantified by qPCR in the sera of IL10^−/−^ mice. (**G**) PCoA of the euclidean distance matrix of miRNA expression in sera from D28 IL10^−/−^ mice (blue), D98 IL10^−/−^ PBS-treated (red) and day 98 IL10^−/−^ anti-TNFα-treated (gray) mice. Percentages on the axes indicate the percentage of variance explained by each component. (**H**,**I)** ratios of colon weights/colon lengths (**H**) and spleen weights (**I**) of mice with the colitic-type signature (dark gray) and non-colitic-type signature (light gray) are displayed as bar graphs. Data are presented as the means +/− S.E.M. (n = 9). Significance was determined using an unpaired t-test (**A,B,C,E,H,I** and **J**) or One-way ANOVA followed by a Bonferroni post-test (**F**) (*p < 0.05; **p < 0.01; ***p < 0.001) compared to the day 98 IL10^−/−^ PBS control group (**A**–**E**) or day 28 IL10^−/−^ control group (**F**).

To further validate the miRNA signature, we lessened the degree of colitis in IL10^−/−^ mice and investigated whether it reflected the treatment of colitis by returning to the non-colitic signature. A previous study showed that the colitic phenotype of IL10^−/−^ mice can be reversed using anti-TNFα antibodies^37^, which is a commonly used therapy in human IBD. The authors of the previous paper used an anti-TNFα antibody dose that had been shown to have a maximum effect in CAIA^38^. Consistent with these prior studies, we treated IL10^−/−^ mice with the anti-murine TNF monoclonal antibody, CNTO5048^39, 40^ (kindly provided by Janssen Biotech, Horsham, PA, USA), at a dosage of 10 mg/kg twice a week starting at D28. To confirm the effect of anti-TNFα therapy in our model, we histologically examined the colon, evaluated the histological scores and assessed various other markers of inflammation. We found that histological scores (Figs 5 and S7A), spleen weight (Fig. 5B), the ratio of colon weight to colon length (Fig. 5C), and fecal Lcn-2 (Fig. 5D) were all decreased in the anti-TNFα-treated group compared to the PBS-treated group. Thus, the anti-TNFα antibody efficiently diminished colitis in the IL10^−/−^ mouse model.

We examined the expression levels of pro-inflammatory cytokines in the colon, and found that the mRNA expression levels of TNFα, interferon-δ (IFN-δ), interleukin-1β (IL-1β) and chemokine (C-X-C motif) ligand 1 (CXCL-1) were decreased in the colons of mice treated with the anti-TNFα antibody, confirming the efficacy of TNFα antagonism in the system (Fig. 5E).

The nine-miRNA signature was then analyzed in the serum of IL10^−/−^ mice treated with or without the anti-TNFα antibody, and compared to that in the D28 non-inflamed control group (Fig. 5F). Overall, the miRNA profile of IL10^−/−^ mice treated with the anti-TNFα therapy was intermediate between those of the non-inflamed group (D28) and the colitis group (D98, PBS treated). The expression levels of miR-29b-3p and miR-122-5p were significantly different between colitic non-treated D98 and anti-TNFα-treated D98 mice, with the latter not significantly different from the non-inflamed group (D28), indicating that anti-TNFα therapy efficiently reversed the expression levels of these two miRNAs (Fig. 5F). No sign of hemolysis was detected in the samples as attested by dCq (miR-23a - miR-451) (Fig. S7B), and the unchanged expression levels of let-7d-3p mmu-miR-21a-5p, two miRNAs that were not identified as colitis-associated, ruled out the possibility of a global circulating miRNA reduction due to anti-TNFα therapy (Fig. S7C and D). To further analyze the reversion of the miRNA signature, we used PCoA. The results showed that of the nine IL10^−/−^ individuals treated with anti-TNFα, five recovered to a “non-inflamed” profile and clustered with the D28 mice (Fig. 5G), whereas the other four animals remained in a cluster with the colitic IL10^−/−^ mice. This is consistent with the observation that reversion of the colitic phenotype was partial and not entirely penetrant (Fig. 5A–E), and that the miRNA signature profile was intermediate between colitic and non-colitic IL10^−/−^ animals (Fig. 5F). Importantly, our analysis of two markers of inflammation (the ratio of colon weight/colon length and spleen weight) revealed that the four mice with colitis-type signatures (1, 4, 5, and 8; Fig. 5G, dark-gray numbers) were more colitic than the five mice (2, 3, 6, 7, 9; Fig. 5G, light gray numbers) harboring non-colitic miRNA signatures (Fig. 5H and I). This difference may be the reflection of a weaker response to anti-TNFα therapy and/or a higher initial level of colitis. Moreover, we observed a correlation between an established marker of intestinal inflammation (CRP) and the principal coordinate 1 of the miRNA signature analysis in anti-TNFα treated IL10^−/−^ mice (R^2^ = 0.5685, P = 0.0189, Fig. 4B), further suggesting that our miRNA signature can be a useful quantitative marker of therapeutic response in IBD.

### The identified miRNA signature can predict UC

**Figure 6:**
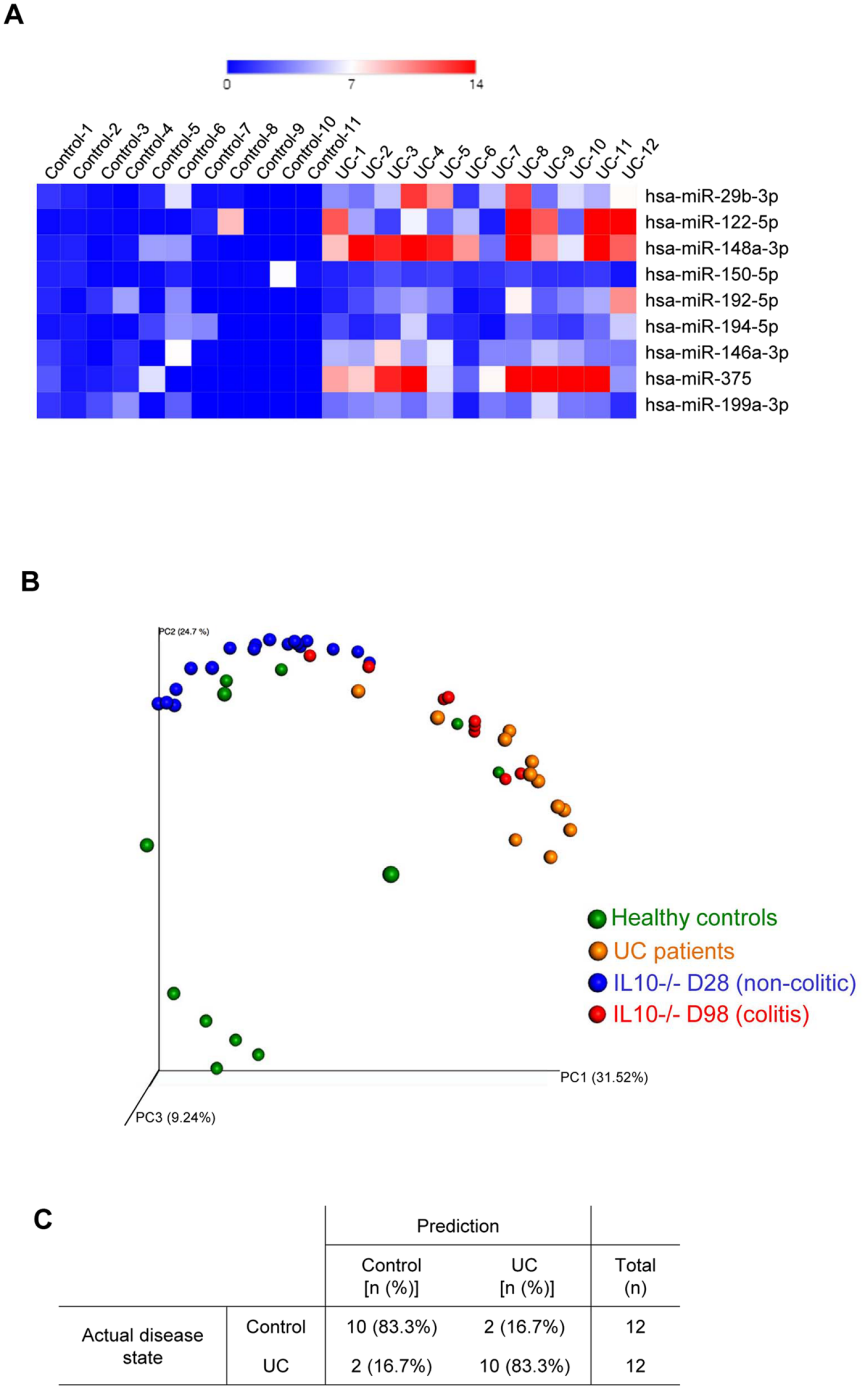
miRNA signature in UC patients. The expression levels of 9 deregulated miRNAs were assesses by qPCR in UC patients. (**A**) Heatmap showing the relative fold changes over controls of the 9 miRNAs in each sample. (**B**) PCoA of the bray curtis distance of miRNA expression in sera from non-colitic mice (in blue), colitic mice (in red), Ulcerative colitis (UC) patients and healthy control. (**C**) kNN (k-Nearest Neighbour) classifier were applied using mice samples (both colitic and non colitic) as discovery cohort and human samples (both UC and controls) as test cohort. Prediction rates of disease state using the previously identified miRNA signature is shown.

We next investigated whether our miRNA signature could be applied to humans for the diagnosis of IBD. We quantified the expression of the previously identified miRNAs in sera from UC patients (Table 1, Fig. 6 and Fig. S8) and examined, based on our mouse PCoA plots, whether they would fall in the region indicating health or disease (Fig. 6). We found that the identified nine-miRNA signature efficiently distinguished ulcerative colitis (UC) patients from normal controls (Table 1, Figs 6A and S8A). In contrast, the commonly used inflammatory markers, CRP and Lcn-2, yielded only modest trends with high inter-individual variations in these same patients (Fig. S8B and C). Importantly, when the mouse and human miRNA expression profiles were compared using a PCoA plot, we observed that all colitic individuals (colitic mice in red and UC humans in orange) formed clusters distinct from those containing the control individuals (non-colitic mice in blue and healthy human controls in green) (Fig. 6B). Next, the k-NN algorithm was used to predict whether the mouse miRNA signature could be used to efficiently discriminate UC patients from controls. Expressions of the nine miRNAs in mice were used as a discovery cohort against the human miRNA expression profiles, in an effort to classify the patients into “ulcerative colitis” and “control” groups. As presented in Fig. 6C, out of 12 controls and 12 UC samples, only two controls and two UC samples were misclassified, for a prediction rate of 83.3%. We thus demonstrated that the disease state of UC patients can be predicted with an accuracy of 83.3% by using their circulating miRNA expression profiles with reference to the mouse dataset as a classifier. We speculate that using a unique human dataset as the classifier would be likely to improve the predictive rate to perhaps near 100%.

**Table 1:** Characteristics of patients with active UC and control.

	Control (n=12)	Ulcerative colitis (n=12)
**Male/Female**	4/8	4/8
**Age (year)**		
Average	49.1	49.1
Range	20–84	20–84
**Medication**		
Mesalamine	—	8
Antibiotics	—	1
Steroids	—	4
Immunomodulators	—	2
Biologics	—	2

These results provide proof of concept for the use of a miRNA signature for IBD diagnosis. We believe that the future development of a high-throughput-identified human dataset will yield a very low misclassification error rate and enable the identification of a powerful, sensitive, specific, and unique IBD-related miRNA signature in humans.

## Discussion

Currently, physicians combine symptom assessment, patient history, physical examination, laboratory and radiological tests, and endoscopic/histological analyses to make the diagnosis, assess the severity, and predict the disease outcome for IBD. Many of these routine procedures are costly and invasive. Moreover, the current molecular markers for IBD (*e.g*., CRP, calprotectin, etc.) lack specificity and sensitivity.

Since the emergence of miRNAs as potential markers for many diseases, studies on miRNAs in IBD have focused on single miRNA and its association with a target gene or group of genes. In particular, miR-143 and miR-145 were found to be down-regulated in the colon biopsies of UC patients^41^, and miR-7 was observed to be down-regulated in the mucosa of CD patients^42^. However, while these miRNAs and their unique targets might be exploited for therapeutic interventions, tissue-extracted miRNAs are not valid biomarkers for IBD. The concept of a circulating miRNA signature has been suggested as being more valuable and practical for various diseases, including IBD. Although relatively few studies have quantitatively assessed circulating miRNAs in patients with IBD, some differences in miRNA expression have been observed in sera from patients with either CD or UC^43–47^. A recent study has identified a set of 4 miRNAs that correlated with UC disease activity with higher specificity and sensitivity than CRP^47^. The potential use of such panels for distinguishing between CD and UC has been described^48^. All these studies demonstrated that it may be possible to monitor miRNA panels in the peripheral blood as a biomarker for IBD.

Herein, we identified in mice a new signature that possesses all of the desired qualities of an ideal biomarker for IBD. In that it: i) distinguishes inflammation of extra-intestinal *versus* intestinal origin; ii) distinguishes various subtypes of intestinal inflammation (IL10^−/−^, DSS, or TLR5^−/−^); iii) reflects the response to therapeutics; and iv) is modified in mice with a genetic predisposition to developing the disease.

We profiled and identified serum miRNAs that were differentially expressed in the sera of IL10^−/−^ mice during the development of colitis. Among the nine validated miRNAs, miR-29b-3p, -122-5p, -192-5p, -194-5p, -375-3p, -150-5p and -146a-3p showed increased expressions, whereas -148a-3p, and -199a-3p showed decreased expressions in IL10^−/−^ colitic *versus* non-colitic mice.

The specificity of our biomarker signature for the presence and types of intestinal inflammation was examined using two other mouse models of colitis (DSS-treated and TLR5^−/−^ mice) and a model of extra-intestinal inflammation (CAIA). It is to be noted that DSS induced-colitis and IL10^−/−^ models represent, respectively, acute and chronic model of intestinal inflammation. The use of a third model of colitis with TLR5^−/−^ mice, which also develop chronic colitis, permitted the comparison between two models of chronic colitis with different origin and severities. We found that most of the miRNAs contained within our signature were specific to the colitis of IL10^−/−^ mice, and were not altered in the other models. However, miR-122-5p and 375-3p were increased in all of the investigated models of inflammation, including arthritis, while miR-150-5p was increased in all of the intestinal-related inflammation models, but not in the arthritic model. The miRNA signature identified herein thus comprises a combination of specific and non-specific markers (Table 2) that we think is giving this signature its unprecedented power and originality. Among the commonly deregulated miRNAs, microRNA-375 was previously described as being up-regulated in serum from UC and CD patients^49^ and in IL10^−/−^ mice^50^. Micro-RNA-122 is known to be increased in several inflammatory conditions^51, 52^, and was recently observed to be increased in uninvolved colonic mucosa of pediatric CD patients^53^ and in colon biopsies during the progression from non-neoplasia to dysplasia in a group of 4 CD patients^54^, and downregulated in transition from dysplasia to cancer in this same group^54^. Finally, miRNA-150 was previously shown to be up-regulated in colonic tissues of DSS-induced colitic mice and UC patients^55^.

**Table 2:** Summary of the miRNA of the signature.

miRNA name	IL10^−/−^ mice model	Intestinal Inflammation	Inflammation
mmu-miR-29b-3p	x		
mmu-miR-122-5p	x	x	x
mmu-miR-148a-3p	x		
mmu-miR-150-5p	x	x	
mmu-miR-192-5p	x		
mmu-miR-194-5p	x		
mmu-miR-146a-5p	x		
mmu-miR-375-3p	x	x	x
mmu-miR-199a-3p	x		

We showed that our nine-miRNA signature could discriminate between the different forms of colitis and arthritis, as well as between non-colitic mice with and without a genetic predisposition to develop the disease (WT mice *versus* non-colitic IL10^−/−^ mice). Whereas the current biomarkers, CRP and Lcn-2, did not discriminate between the DSS-induced and IL10^−/−^ mouse models of colitis, our signature could make this distinction. Moreover, our miRNA signature reflected the therapeutic response of IL10^−/−^ mice to anti-TNFα therapy, discriminating between mice that did and did not show a therapeutic response.

Our panel was then validated using sera from patients with UC. Our results showed that the miRNA signature identified in mice could predict the disease status in patient’s samples. This crucial result supports the concept of using a miRNA signature for IBD diagnostics. It was established that UC differed from CD in that UC seemed to be a Th2 cytokine-mediated disease whereas CD seemed to be Th1 cytokine-mediated^56^. Since our miRNA signature was first identified in IL10^−/−^ mice characterized by activation of TH1 cells, hence mimicking CD with that regard, a high throughput approach need to be done in both UC and CD patients.

Regarding the outcomes of the anti-TNFα therapy in IL10^−/−^ mice, the miRNA expression profile was not fully reversed when the inflammation was lowered. Particularly, miR-375-3p and miR-199a-3p had similar expression levels in D98 anti-TNFα treated group and non-treated group. One possible explanation is that for these two specific miRNAs, inflammation is not the main trigger which leaves other causes to be speculated, such as a possible specificity of these two miRNAs to the genetic susceptibility (IL10^−/−^). The question of the reversibility of the signature could also be investigated in other models of colitis with other etiologies (infectious colitis for example).

Future studies should also focus on defining the cells secreting specific serum miRNAs and investigate their physiological relevance. This will help to determine whether serum miRNAs are passive molecules secreted upon intestinal inflammation or act as key players to communicate a state of distress that occurs in the intestine^57^. Indeed, several groups have reported identification of circulating miRNAs found in exosomes as a form of cell-to-cell communication^58^.

Other studies investigated the concept of miRNA signature for IBD in patient groups (UC and CD *vs*. healthy patients)^43, 44, 49^. Here, we used mouse models that facilitate longitudinal analyses and give access to a large number of samples, a uniform genetic background, a controlled environmental exposure, and uniform sample collection. None of the miRNAs identified in our study were part of the miRNA panels previously identified in sera of IBD patients. While mice and human share identical miRNAs, identifying different miRNA panels in our present work performed longitudinally in mice compared to previously reported ones in patients compared to healthy control subjects, was expected.

Given that sera contain only a limited number of miRNAs, profiling used here was limited to 138 miRNAs known to be present in the serum. This number is in the range of previously published studies using serum material^59, 60^. However, some miRNAs potentially deregulated in colitis that are not included with this approach could be missed. In the future, a full miRNome profiling of sera from IBD patients enrolled in a longitudinal study and validated in humans with non-IBD intestinal inflammation (e.g. celiac disease) will be needed to enable refining the signature and establish a panel that shows the greatest clinical diagnostic value. Similarly to what we have described in mice, such a panel in human should be proving its sensitivity, specificity and potency for early diagnosis, while distinguishing different subtypes of IBD and monitoring the therapeutic response.

This study, is a proof of principle that miRNA signatures could be an innovative, specific and reliable diagnostic approach to overcome frequent misdiagnoses in IBD and numerous diseases. Importantly, a miRNA signature could be assessed on the individual level using a readily obtained sample type (blood). Effective methodologies to perform direct quantification of absolute miRNAs in a reliable and simple manner amenable to the point-of-care testing are needed. As a result of such need, a significant progress has been made toward this goal with the development of a microelectrode miRNA sensor that allows for one-step detection of miRNAs within 10 min^61^.

## Methods

### Mice

Three week-old female C57BL/6 wild type (WT) and IL-10^−/−^ mice were obtained from Jackson Laboratories (Bar Harbor, ME). TLR5^−/−^ mice were grown in our facility. Mice were group housed under a controlled temperature (25 °C) and photoperiod (12:12-h light–dark cycle) and fed *ad libitum*. Animal experiments were approved by the Institutional Animal Care and Use Committee of Georgia State University (Atlanta, GA), and performed in accordance with the guide for the Care and Use of Laboratory Animals by U.S. Public Health Service. All procedures were approved and registered in the protocol IACUC IDs: A14007 and A14010.

### Development of colitis in IL10^−/−^ and TLR5^−/−^ mice

IL-10^−/−^ mice develop colitis on a time dependent manner. In order to assess the intestinal inflammation in those mice at different time of colitis development, feces were collected from day 42 to day 128 and at different time points to measure Lcn-2. Two hundred microliters of blood were collected in retro-orbital in serum separator tubes (BD Biosciences, Franklin Lakes, NJ) at day 30, 58, 86, 114, 128 to obtain sera and analyze miRNA profiles. TLR5^−/−^ mice develop colitis in a subset of mice. In order to identify the mice with colitis Lcn-2 was measured in serum and threshold was defined as 150 ng/ml.

### Anti-TNFα antibody treatment of IL10^−/−^ mice

IL10^−/−^ mice were treated twice a week from 4 weeks of age (28 days) to 14 weeks (98 days) by intraperitoneal (IP) injection of equal volumes of either anti-murine TNFα antibody (10 mg/kg, 200–250 μg, CNTO5048, kindly provided by Janssen Biotech, Horsham, PA, USA) or PBS. Retro-orbital blood collection (200 µl) was performed every two weeks from 4-weeks to 14-weeks to obtain the serum and measure the miRNAs at these different time points. Mice were euthanized at day 98 and spleen weights, colon weights and colon lengths were measured. Organs were collected for downstream analysis.

### DSS induced colitis

C57BL/6 WT mice were administered DSS (MP Biomedicals, Solon, OH) at 3% in drinking water ad libitum for 7 days. Feces and 100 µl of blood were collected at day 0 (before DSS treatment) and day 7. Sera were collected by centrifugation using serum separator tubes. Mice were sacrificed by CO_2_ euthanasia.

### Collagen Antibody-Induced Arthritis Model

BALB/C WT mice received collagen antibodies injections (200 µL) on day 0 by an intravenous injection (tail vein). On day 6, mice received a LPS boost injection (200 µL) by intraperitoneal injection. Assessment of the animals was performed on pretreatment (day-2), and on day 12. The disease severity scores was based on the following scoring 0 to 4: (0) normal, (1) erythema and mild swelling confined to the mid-foot (tarsals) or ankle joint, or digits, (2) erythema and mild swelling extending from the ankle to the mid-foot (2 segments), (3) erythema and moderate swelling extending from the ankle to the metatarsal joints (2 segments), (4) erythema and severe swelling encompassing the ankle, foot, and digits. Blood samples (100 µl) were collected from each mouse on pretreatment (day -2) and on day 12 from the jugular vein. Sera were collected by centrifugation using serum separator tubes.

### H&E staining of colonic tissue

Mouse colons were fixed in 10% buffered formalin for 24 hours at room temperature and then embedded in paraffin. Tissues were sectioned at 5-μm thickness and stained with hematoxylin & eosin (H&E) using standard protocols. Images were acquired using a Zeiss Axioskop 2 plus microscope (Carl Zeiss MicroImaging) equipped with an AxioCam MRc5 CCD camera (Carl Zeiss). For the anti-TNFα experiment, each colon was assigned four scores based on the degree of epithelial damage and inflammatory infiltrate in the mucosa, submucosa and muscularis/serosa, as previously described^62^. A slight modification was made to this scoring system; each of the four scores was multiplied by 1 if the change was focal, 2 if it was patchy and 3 if it was diffuse, as previously described^13^. The 4 individual scores per colon were added, resulting in a total scoring range of 0–36 per mouse. The scores of each mice within each group were averaged.

### Quantification of fecal and serum Lcn-2 and serum CRP by ELISA

Fecal samples were reconstituted in PBS containing 0.1% Tween 20 (100 mg/ml). After centrifugation, clear supernatants were collected. Serum samples were diluted in kit-recommended reagent diluent (1.0% BSA in PBS). Murine or human Lcn-2 and CRP levels were estimated in the supernatants and/or serum using Duoset murine/human Lcn-2 and CRP ELISA kits (R&D Systems, Minneapolis, MN).

### Colonic mRNA expression analysis by qPCR

RNA Extraction and Real-Time RT-PCR Total RNA were extracted from colonic tissues using RNeasy mini Kit (Qiagen) according to the manufacturer’s instructions. Yield and quality of RNA were verified with a Synergy 2 plate reader (BioTek, Winooski, VT, USA). cDNA were generated from the total RNA isolated above using the Maxima first-strand cDNA synthesis kit (Thermo Scientific, Lafayette, CO, USA). mRNA expression were quantified by quantitative real-time reverse transcription-PCR (qRT-PCR) using Maxima SYBR green quantitative PCR (qPCR) Master Mix (Thermo Scientific) and the following sense and antisense primers: TNF α 5′-AGGCTGCCCCGACTACGT-3′ and 5′-GACTTTCTCCTGGTATGAGATAGCAAA-3′; IFN-δ 5′-CAGCAACAGCAAGGCGAAA-3′ and 5′-CTGGACCTGTGGGTTGTTGAC-3′; IL-1β 5′-TCGCTCAGGGTCACAAGAAA-3′ and 5′-CATCAGAGGCAAGGAGGAAAAC-3′; CXCL-1 5′-TTGTGCGAAAAGAAGTGCAG-3′ and 5′-TACAAACACAGCCTCCCACA-3′; 36B4 5′-TCCAGGCTTTGGGCATCA-3′ and 5′-CTTTATCAGCTGCACATCACTCAGA-3′. Results were normalized by using 36B4 housekeeping gene.

### miRNA profiling

miRCURY LNA Universal RT miRNA PCR Human serum/plasma panel, using a mouse annotation containing 138 assays was used to profile miRNAs differentially expressed in IL10^−/−^ mouse serum at different ages, 30 days, 58 days, 86 days, 114 days and 128 days (Exiqon). PCR reactions that gave rise to multiple melting curve peaks or single peaks with unexpected melting temperature were removed from the dataset. Normalization of data was performed using the global mean. All data was normalized to the average of 104 miRNAs detected in all samples (average – assay Cp). RNA spike-in control (UniSp6) was used to test the efficiency of the complementary DNA synthesis reaction, whereas DNA spike-in control (UniSp3) tested the efficiency of qPCR amplification.

### Assessment of hemolysis

Hemolysis was detected using the ratio of miR-451a (a miRNA highly expressed in red blood cells) to miR-23a-3p (a microRNA unaffected by hemolysis) a sensitive indicator detecting down to 0.001% hemolysis in serum^32^. Delta Cq (miR-23a-3p - miR- 451a) were measured and possible erythrocyte microRNA contaminations were indicated by Delta Cq ≥ 5, Delta Cq ≥ 7–8 or more indicated a high risk of hemolysis^33^.

### miRNA expression analysis by qPCR

Total RNAs were extracted from serum samples using miRCUCY RNA Isolation kit-biofluids (Exiqon) according to the manufacturer’s instructions. Ten nanograms of total RNAs was reversed transcribed using miRCURY LNA Universal RT microRNA PCR protocol using the Universal cDNA synthesis kit II (Exiqon). cDNA were diluted 1:50 and assayed in 10 µL PCR reactions using custom design Pick & Mix microRNA PCR panels and the ExiLENT SYBR Green master mix following the instruction for miRCURY LNA Universal RT microRNA PCR. Each of the miRNA listed in Table S2 was assayed once by qPCR. RNA spike-in control (UniSp6) was used for normalization. Pick-N-Mix qPCR plates were run on RealPlex mastercycler (Eppendorf). All reagents used for quantitative PCR were obtained from Exiqon.

### UC patients’s serum samples

Serum samples from active ulcerative colitis and gender- and aged-matched normal control subjects were purchased from Boston Biosource (Newton, MA). Blood was collecting from patients or healthy control in serum separator tubes to obtain serum. The following exclusion criteria applied for the selection of patients: diabetes, auto-immune diseases, cardiovascular diseases, all cancers, human immunodeficiency virus, hepatitis B and C viruses or all other infectious diseases, pregnancy and all other diseases. The characteristics of the patients are detailed in Table 1.

### Data analysis

QIIME software was used to analyze the beta-diversity of the Euclidean distance, with principal coordinates subsequently calculated. Emperor software was used to make the 3D-plots. Principal coordinates analysis (PCoA) plots were drafted in order to assess variation between experimental groups. For PCoA compiling mice and human data (Fig. 6A), average of control groups (non colitic and human control) were defined as 1 in order to normalize the variation of expression inherent to the samples differences (species, collection…). The expression levels of non-detectable miRNAs were defined as 0. Heat map were generated using matrix visualization GENE-E software with Unsupervised Hierarchical Clustering to visualize the expression patterns of each miRNA. A relative color scheme that uses the minimum and maximum values in each row to convert values to colors was represented. The R software and PamR package were used in order to draw misclassification error rate graphical representation (Fig. S8B). The R software and kNN (k-Nearest Neighbors) algorithm (class package) were used to predict whether mice miRNA signature can efficiently discriminate UC-patients from non-UC patients. The expression of the 9 miRNA in mice was used as a discovery cohort, and applied to the human miRNA expression in order to classify the patient in “UC” and “non-UC” groups.

### Statistical analysis

Prior to determining the significance with parametric tests, normality was tested using D’Agostino & Pearson omnibus test. For normally distributed samples, significance was determined using either unpaired two-tailed Student’s t-test or one-way ANOVA followed by a Bonferroni post-test. Mann-Whitney test or Kruskal-Wallis test followed by a Dunn’s post-test were used when samples failed the normality test (GraphPad Prism software). Histograms are represented with standard error of the mean in order to report the precision of the mean. Differences were noted as significant *p < 0.05, **p < 0.01 and ***p < 0.001. For principal coordinates analysis, PERMANOVA tests were used to determine the significance of clustering occurring between experiment groups.

## Electronic supplementary material

Supplementary info
