# Supplementary material for: Serum miRNA signature diagnoses and discriminates murine colitis subtypes and predicts ulcerative colitis in humans
Source: Sci Rep. 2017 May 31;7:2520. doi: 10.1038/s41598-017-02782-1 (PMC5451415; doi:10.1038/s41598-017-02782-1)
Supplement: Supplementary file 1 — Supplementary info [file 41598_2017_2782_MOESM1_ESM.pdf]

Serum miRNA signature diagnoses and discriminates murine colitis subtypes and predicts ulcerative colitis in humans

Emilie Viennois[1*], Yuan Zhao[1, 2], Moon Kwon Han[1], Bo Xiao[1, 3], Mingzhen Zhang[1], Meena Prasad[4, 5], Lixin Wang[1, 4], Didier Merlin[1, 4].

[1] Institute for Biomedical Sciences, Center for Diagnostics and Therapeutics, Georgia State University, Atlanta, GA 30303, USA.

[2] Department of Gastroenterology, Zhongshan Hospital, Fudan University, Shanghai, China.

[3] Institute for Clean Energy and Advanced Materials, Faculty for Materials and Energy, Southwest University, Chongqing, 400715, China.

[4] Veterans Affairs Medical Center, Decatur, GA, USA.

[5] Emory University, Department of Medicine, Atlanta, GA, USA

*Author for correspondence

Tel.: +1 (404) 413 3598

Fax: +1 (404) 413 3580

Email: eviennois@gsu.edu

Fig. S1: Spontaneous development of colitis in IL10$^{-/-}$ mice. A, WT and IL-10$^{-/-}$ 135-day-old mice were subjected to H & E staining of colon samples. Arrowheads indicate lymphocyte infiltration. **B,** Lcn-2, which is used as a marker of intestinal inflammation, was monitored from day 40 to day 127 by ELISA (n=6-7).

Fig. S2: Heat map and unsupervised hierarchical clustering on the 50 miRNAs with highest standard deviations. Total RNAs from serum collected from 30 animals was used for miRNA profiling. Clustering was performed on all 30 samples, using the top 50 miRNAs with highest standard deviations. The normalized (dCp) values were analyzed.

Fig. S3: Number of detected samples and quality control of the high throughput approach. A, Number of miRNAs detected per sample (count) and their average amplification threshold (Cp) values in each sample. **B,** Raw Cp values obtained from the two controls, UniSp6 and UniSp3, which were used to test the assay performance for each sample. **C,** Hemolysis was assessed using the ratio of miR-451a to miR-23a-3p. Possible erythrocyte microRNA contaminations were indicated by a Delta Cq (miR-23a-3p - miR-451a) ≥ 5 (orange area). Delta Cq ≥ 7–8 or more indicated a high risk of hemolysis (red area).

Fig. S4: Assessment of inflammation in various mouse models. The degree of inflammation was assessed in arthritic mice **(A)**, DSS-treated mice **(B)** and TLR5$^{-/-}$ mice **(C)**. **A,** The levels of Lcn-2 were measured in the sera obtained from CAIA mice at D12 (after induction of arthritis) and in the same mice at D2 (before induction of arthritis). Histological scores were displayed **(A)**. **B,** The levels of Lcn-2 were measured in the feces of WT mice after 7 days (D7) of

exposure to 3% DSS in the drinking water, and compared to the levels obtained from the same mice at D0. **C,** The levels of Lcn-2 were measured in the sera of TLR5$^{-/-}$ mice and corresponding WT controls. Significance was determined using either an unpaired t-test or Mann Whitney test if the concentration values did not follow a normal distribution **(B)** (*, p<0.05; and **, p<0.01) and compared to the corresponding non-inflamed control. **D,** Hemolysis was assessed using the ratio of miR-451a to miR-23a-3p expression levels in sera arthritic and its controls (D12 and D-2 respectively), DSS-treated and water-control (WT-DSS-D7 and WT-D0 respectively) and TLR5-/- and its corresponding WT controls mice. Possible erythrocyte microRNA contaminations were indicated by a Delta Cq (miR-23a-3p - miR-451a) ≥ 5 (orange area).

Fig. S5: Lcn-2 levels in the sera of various mouse model of inflammation. Lcn-2 levels were measured in the sera of WT, D7 DSS, TLR5$^{-/-}$, D28 IL10$^{-/-}$, D98 IL10$^{-/-}$ and arthritic mice. Data were presented as the means +/- S.E.M. (n=5-18). Significance was determined using Kruskal-Wallis followed by a Dunn's multiple comparison post-test (*, p<0.05; and **, p<0.01).

Fig. S6: miRNA signature in the context of cage effect and genetic background. PCoA of the euclidean distance matrix of the expression of the nine signature miRNAs in sera of WT mice hosted in four different cages **(A)** or representing two different genetic backgrounds, Bab/C or C57/Bl5 (cage A or cage B) **(B)**.

Fig. S7: Effect of Anti-TNFα treatment on IL10-/- mice. A, Colon section were stained by H&E, scale bar=100µm. **B,** Hemolysis was assessed using the ratio of miR-451a to miR-23a-3p expression levels in sera of D28 IL10-/-, D98 IL10$^{-/-}$ PBS-treated and day 98 IL10$^{-/-}$ anti-TNFα-

treated mice. Possible erythrocyte microRNA contaminations were indicated by a Delta Cq (miR-23a-3p - miR-451a) ≥ 5 (orange area). The serum expression levels of let-7d-3p **(C)** and mmu-miR-21a-5p **(D)** were compared between D28 IL10$^{-/-}$ mice, D98 IL10$^{-/-}$ PBS-treated and day 98 IL10$^{-/-}$ anti-TNFα-treated mice and expressed as Fold change over Day 28 control. Significance was tested using Kruskal-Wallis followed by a Dunn's multiple comparison post-test.

Fig. S8: miRNA signature in IBD patients. The expression levels of the 9 deregulated miRNAs pertaining to the signature identified in mice were quantified by qPCR in sera of patients with ulcerative colitis (UC) and controls. **A,** Overall misclassification error rate using from 1 (right) to 9 (left) miRNAs of the signature panel. **B-C,** CRP (**B**) and Lcn-2 (**C**) levels were measured by ELISA in the sera of control and ulcerative colitis patients. Data were presented as the means +/- S.E.M. (n=11). Significance was evaluated using either an unpaired t-test **(B)** or Mann Whitney test when the concentration values did not follow a normal distribution **(C)**.

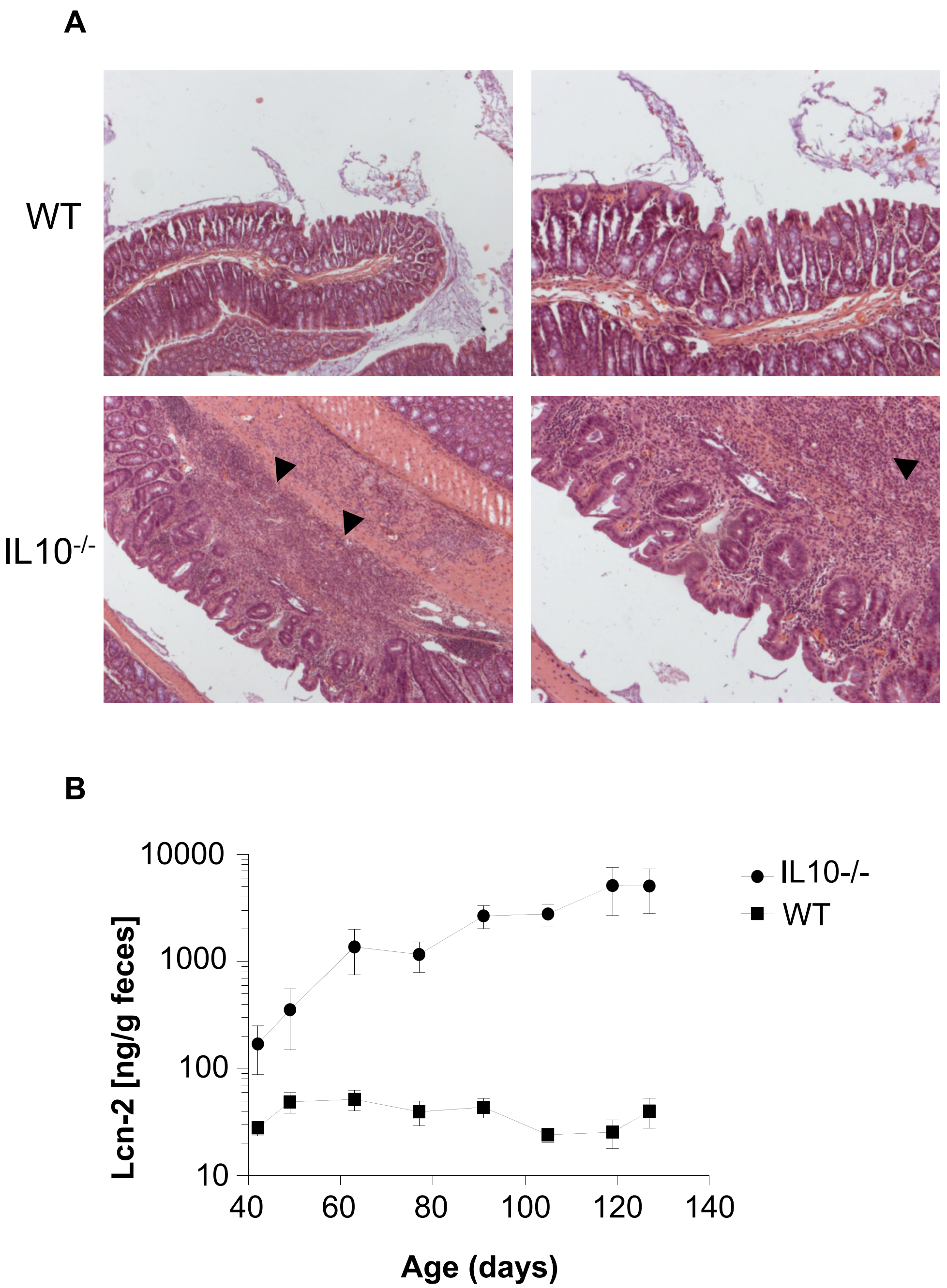

A
WT
IL10-/-
B
10000
1000
100
10
Lcn-2 [ng/g feces]
40
60
80
100
120
140
Age (days)
IL10-/-
WT

Fig. S1

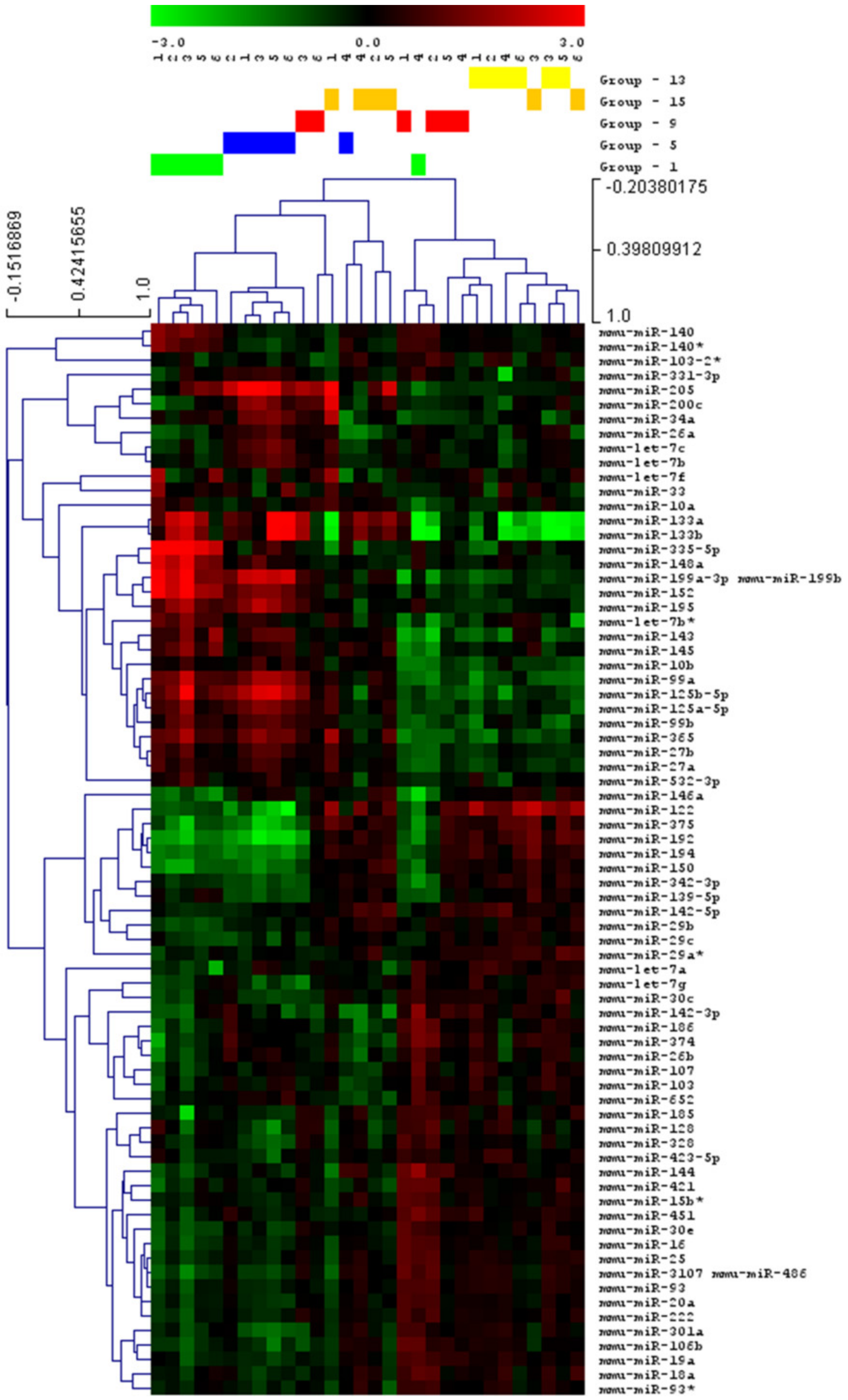
-3.0
0.0
3.0
Group - 13
Group - 15
Group - 9
Group - 5
Group - 1
-0.20380175
0.39809912
1.0
-0.1516869
0.42415655
1.0
mmu-miR-140
mmu-miR-140*
mmu-miR-103-2*
mmu-miR-331-3p
mmu-miR-205
mmu-miR-200c
mmu-miR-34a
mmu-miR-26a
mmu-let-7c
mmu-let-7b
mmu-let-7f
mmu-miR-33
mmu-miR-10a
mmu-miR-133a
mmu-miR-133b
mmu-miR-335-5p
mmu-miR-148a
mmu-miR-199a-3p mmu-miR-199b
mmu-miR-152
mmu-miR-195
mmu-let-7b*
mmu-miR-143
mmu-miR-145
mmu-miR-10b
mmu-miR-99a
mmu-miR-125b-5p
mmu-miR-125a-5p
mmu-miR-99b
mmu-miR-365
mmu-miR-27b
mmu-miR-27a
mmu-miR-532-3p
mmu-miR-146a
mmu-miR-122
mmu-miR-375
mmu-miR-192
mmu-miR-194
mmu-miR-150
mmu-miR-342-3p
mmu-miR-139-5p
mmu-miR-142-5p
mmu-miR-29b
mmu-miR-29c
mmu-miR-29a*
mmu-let-7a
mmu-let-7g
mmu-miR-30c
mmu-miR-142-3p
mmu-miR-186
mmu-miR-374
mmu-miR-26b
mmu-miR-107
mmu-miR-103
mmu-miR-652
mmu-miR-185
mmu-miR-128
mmu-miR-328
mmu-miR-423-5p
mmu-miR-144
mmu-miR-421
mmu-miR-15b*
mmu-miR-451
mmu-miR-30e
mmu-miR-16
mmu-miR-25
mmu-miR-3107 mmu-miR-486
mmu-miR-93
mmu-miR-20a
mmu-miR-222
mmu-miR-301a
mmu-miR-106b
mmu-miR-19a
mmu-miR-18a
mmu-miR-93*

Fig. S2

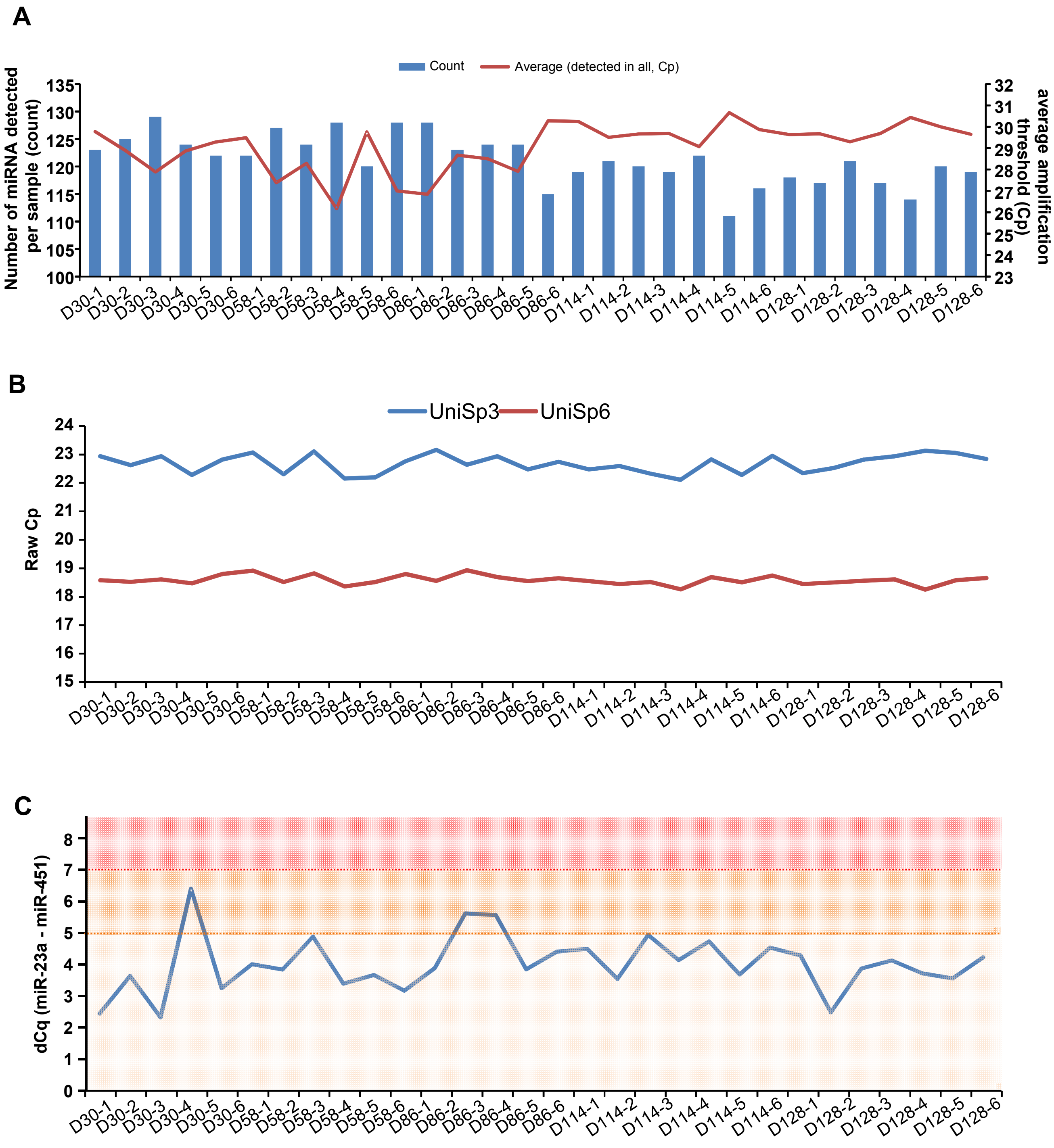

A
Count
Average (detected in all, Cp)
Number of miRNA detected per sample (count)
average amplification threshold (Cp)
B
UniSp3
UniSp6
Raw Cp
C
dCq (miR-23a - miR-451)

Fig. S3

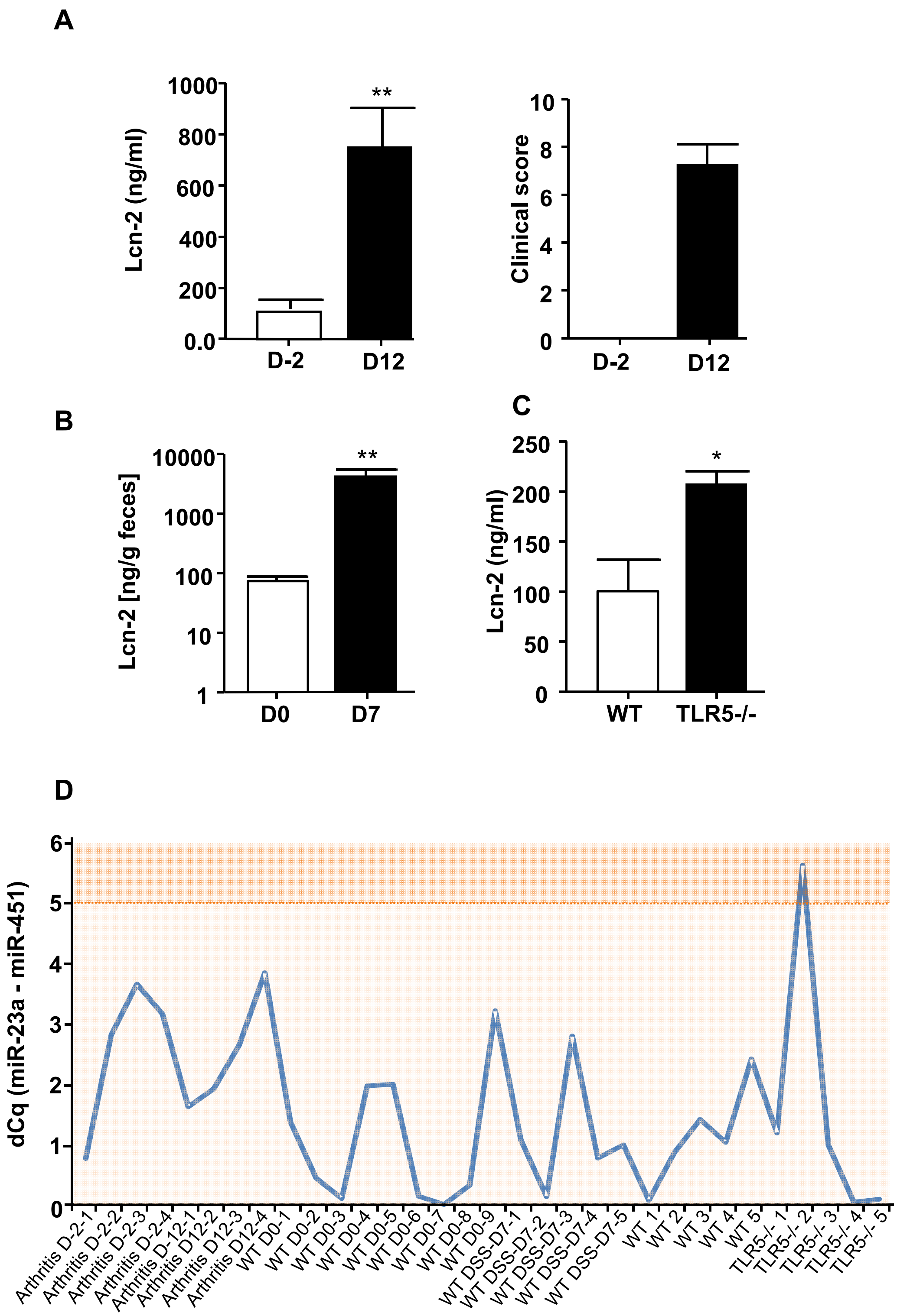
A
Lcn-2 (ng/ml)
1000
800
600
400
200
0.0
**
D-2
D12
Clinical score
10
8
6
4
2
0
D-2
D12
B
Lcn-2 [ng/g feces]
10000
1000
100
10
1
**
D0
D7
C
Lcn-2 (ng/ml)
250
200
150
100
50
0
*
WT
TLR5-/-
D
dCq (miR-23a - miR-451)
6
5
4
3
2
1
0
Arthritis D-2-1
Arthritis D-2-2
Arthritis D-2-3
Arthritis D-2-4
Arthritis D-12-1
Arthritis D12-2
Arthritis D12-3
Arthritis D12-4
WT D0-1
WT D0-2
WT D0-3
WT D0-4
WT D0-5
WT D0-6
WT D0-7
WT D0-8
WT D0-9
WT DSS-D7-1
WT DSS-D7-2
WT DSS-D7-3
WT DSS-D7-4
WT DSS-D7-5
WT 1
WT 2
WT 3
WT 4
WT 5
TLR5-/- 1
TLR5-/- 2
TLR5-/- 3
TLR5-/- 4
TLR5-/- 5

Fig. S4

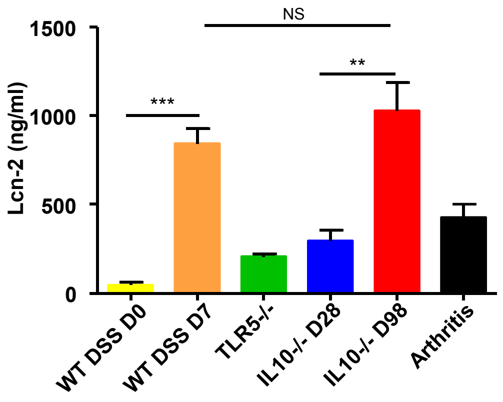
NS
**
***
1500
1000
500
0
Lcn-2 (ng/ml)
WT DSS D0
WT DSS D7
TLR5-/-
IL10-/- D28
IL10-/- D98
Arthritis

Fig. S5

A

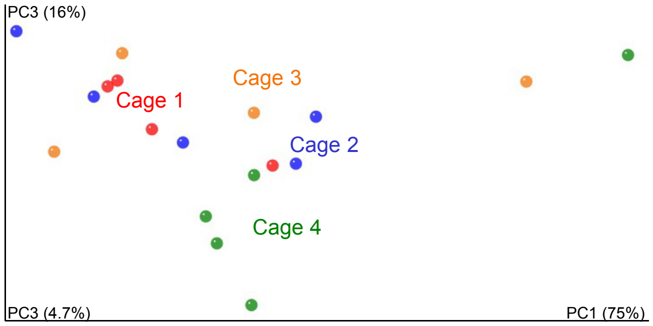
PC3 (16%)
Cage 3
Cage 1
Cage 2
Cage 4
PC3 (4.7%)
PC1 (75%)

B

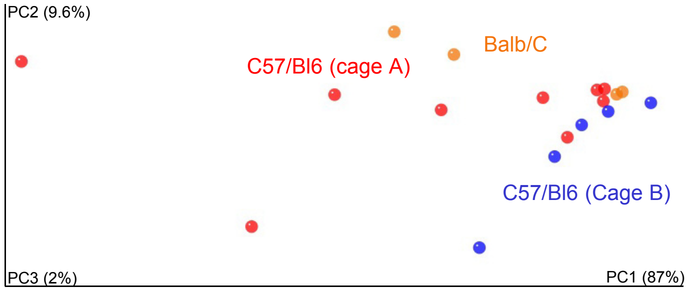
PC2 (9.6%)
Balb/C
C57/Bl6 (cage A)
C57/Bl6 (Cage B)
PC3 (2%)
PC1 (87%)

Fig. S6

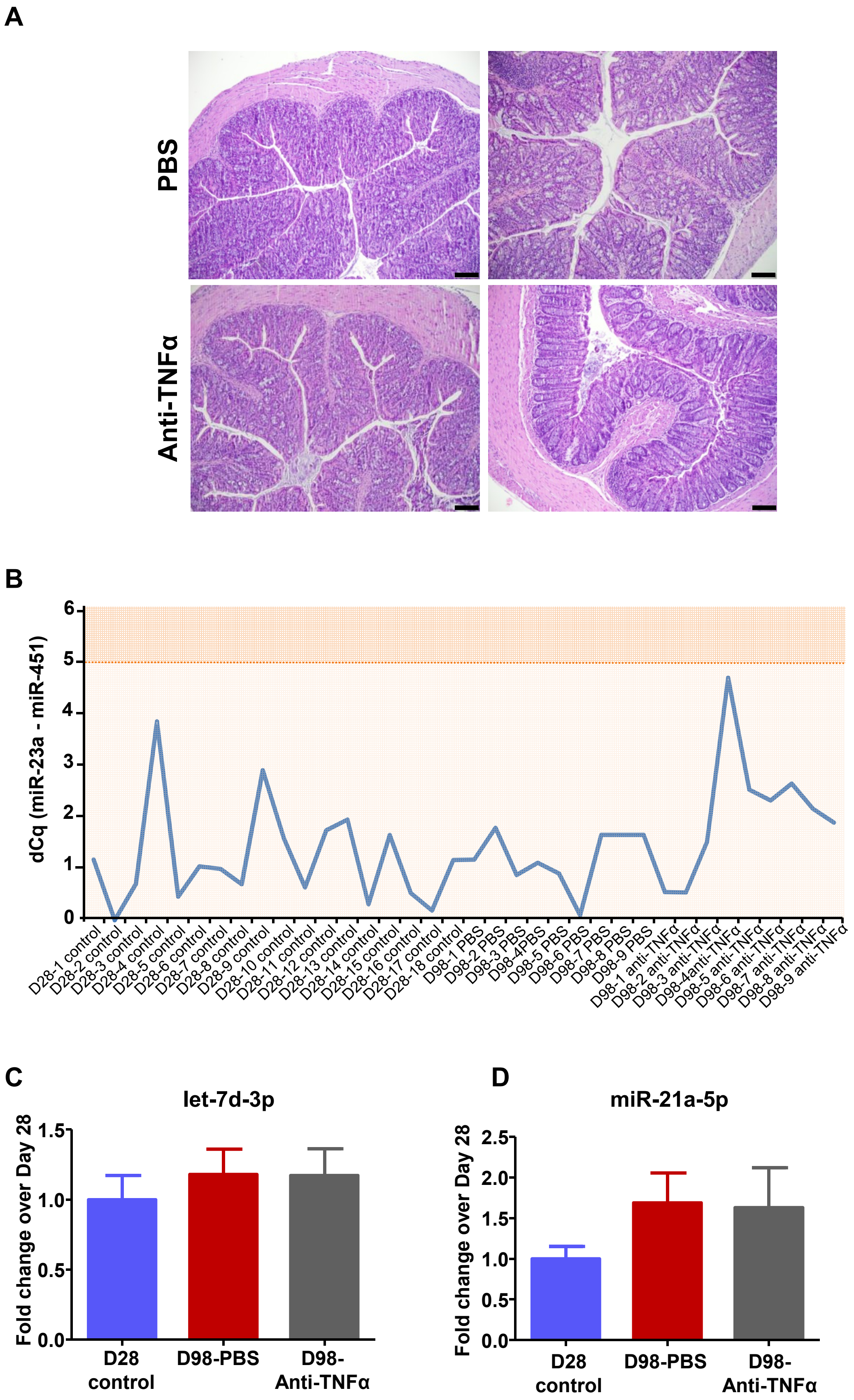
A
PBS
Anti-TNFα
B
dCq (miR-23a - miR-451)
0
1
2
3
4
5
6
D28-1 control
D28-2 control
D28-3 control
D28-4 control
D28-5 control
D28-6 control
D28-7 control
D28-8 control
D28-9 control
D28-10 control
D28-11 control
D28-12 control
D28-13 control
D28-14 control
D28-15 control
D28-16 control
D28-17 control
D28-18 control
D98-1 PBS
D98-2 PBS
D98-3 PBS
D98-4PBS
D98-5 PBS
D98-6 PBS
D98-7 PBS
D98-8 PBS
D98-9 PBS
D98-1 anti-TNFα
D98-2 anti-TNFα
D98-3 anti-TNFα
D98-4anti-TNFα
D98-5 anti-TNFα
D98-6 anti-TNFα
D98-7 anti-TNFα
D98-8 anti-TNFα
D98-9 anti-TNFα
C
let-7d-3p
Fold change over Day 28
0.0
0.5
1.0
1.5
D28 control
D98-PBS
D98-Anti-TNFα
D
miR-21a-5p
Fold change over Day 28
0.0
0.5
1.0
1.5
2.0
2.5
D28 control
D98-PBS
D98-Anti-TNFα

Fig. S7

A

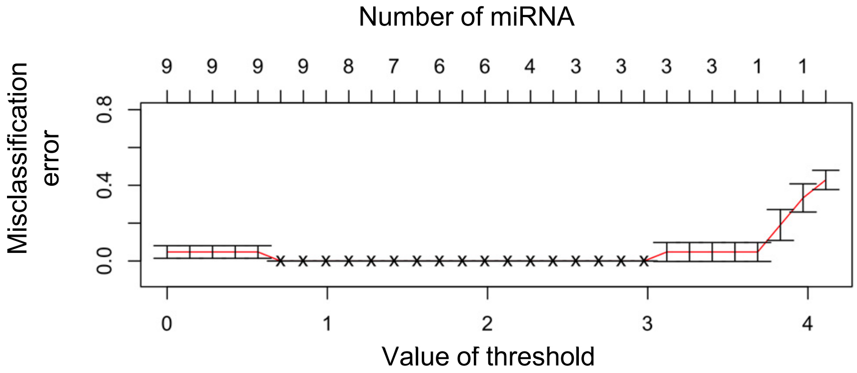
Number of miRNA
9 9 9 9 8 7 6 6 4 3 3 3 3 1 1
Misclassification error
0.0
0.4
0.8
0 1 2 3 4
Value of threshold

B

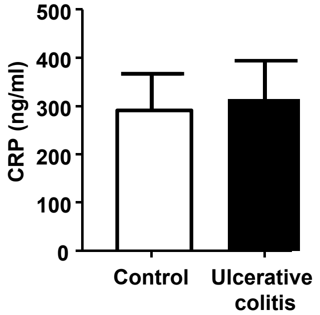
CRP (ng/ml)
0
100
200
300
400
500
Control
Ulcerative colitis

C

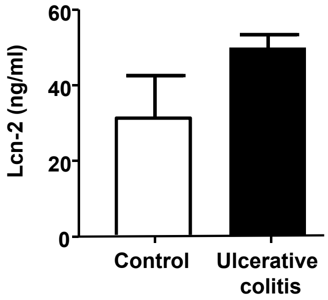
Lcn-2 (ng/ml)
0
20
40
60
Control
Ulcerative colitis

Fig. S8

Table S1: Differentially expressed miRNAs

miRNA name	p-value	Day 30	Day 58	Day 86	Day 144	Day 128
mmu-miR-29b-3p	2.20984E-11	-1.85678	-1.31101	-1.07452	-0.89689	-0.56256
mmu-miR-122-5p	3.48817E-10	-1.66072	-2.39277	-0.55322	0.859965	0.754249
mmu-miR-335-5p	1.94867E-09	-2.52565	-4.8514	-5.36298	-5.66157	-5.71652
mmu-miR-148a-3p	5.24761E-09	0.843239	-0.61949	-1.25158	-1.15293	-1.26707
mmu-miR-150-5p	1.5041E-08	2.226064	2.689313	3.604217	3.997394	4.171364
mmu-miR-192-5p	1.7257E-08	-0.17312	-0.4125	1.149	2.230886	2.231184
mmu-miR-152-3p	6.18033E-08	0.787775	-0.2233	-1.63743	-1.74777	-1.69626
mmu-miR-140-3p	6.94695E-08	1.766723	0.387534	0.782188	0.557419	0.415307
mmu-miR-194-5p	8.96751E-08	-4.11013	-4.1322	-3.14622	-2.43495	-2.22487
mmu-miR-331-3p	2.52224E-07	-3.72845	-3.25325	-3.76908	-4.13136	-4.07768
mmu-miR-195a-5p	4.98283E-07	0.35586	0.493485	-0.81792	-1.12183	-1.13365
mmu-miR-140-3p	9.1114E-07	4.24657	2.950387	3.446355	3.33107	2.894692
mmu-miR-146a-5p	9.44207E-07	-2.78755	-1.24328	-1.82942	-1.12107	-1.00851
mmu-miR-375-3p	1.44853E-06	-2.68252	-2.70641	-1.44698	-0.68609	-0.64424
mmu-miR-199a-3p	2.12411E-06	1.057058	0.97391	-1.56044	-1.64845	-1.0376
mmu-miR-142-5p	1.07633E-05	-1.52849	-1.34446	-1.10408	-0.73419	-0.65949
mmu-miR-29a-3p	1.46988E-05	0.759489	0.873368	1.152408	1.484208	1.548141
mmu-miR-125b-5p	1.7419E-05	1.939287	2.585686	0.412086	-0.11031	0.578385
mmu-miR-154-5p	3.9972E-05	-3.83244	-3.72537	-6.29341	-5.94904	-5.15702
mmu-miR-22-5p	4.42778E-05	-2.52429	-2.99868	-3.01969	-2.88556	-2.7906
mmu-miR-378a-5p	5.31944E-05	0.125588	-0.08127	0.247624	0.344823	0.346599
mmu-miR-29c-3p	6.99251E-05	-0.05319	0.041435	0.367293	0.706166	0.727192
mmu-miR-99a-5p	8.0944E-05	-0.20936	0.171501	-1.55012	-2.06515	-1.31727
mmu-miR-342-3p	9.96511E-05	0.444511	0.419141	0.728975	1.462688	1.481494
mmu-miR-30d-5p	0.000101673	-0.60119	-0.45227	-0.34462	-0.54416	-0.51075
mmu-miR-29a-5p	0.000116278	-2.98111	-2.80937	-2.7168	-2.11207	-2.34437
mmu-miR-132-3p	0.000163984	-2.52996	-3.0935	-2.46304	-2.16142	-2.43404
mmu-miR-143-3p	0.000181148	0.120318	0.153203	-1.4093	-1.33167	-0.41832
mmu-miR-423-3p	0.000184882	-0.50883	-1.1901	-0.86712	-0.73864	-0.80031
mmu-miR-99b-5p	0.000264048	-1.97351	-1.82111	-2.74548	-3.10603	-2.50506
mmu-miR-365-3p	0.000273826	-1.08254	-0.85033	-2.46115	-2.71846	-1.64456
mmu-miR-328-3p	0.000434575	1.825247	1.276928	2.36195	1.97603	1.576135

Table S2: miRNA sequences

miRNA name	miRNA sequence 5'-3'
mmu-miR-29b-3p	TAGCACCATTTGAAATCAGTGTT
mmu-miR-122-5p	TGGAGTGTGACAATGGTGTTTG
mmu-miR-335-5p	TCAAGAGCAATAACGAAAAATGT
mmu-miR-148a-3p	TCAGTGCACTACAGAACTTTGT
mmu-miR-150-5p	TCTCCCAACCCTTGTACCAGTG
mmu-miR-192-5p	CTGACCTATGAATTGACAGCC
mmu-miR-140-5p	CAGTGGTTTTACCCTATGGTAG
mmu-miR-194-5p	TGTAACAGCAACTCCATGTGGA
mmu-miR-195a-5p	TAGCAGCACAGAAATATTGGC
mmu-miR-140-3p	TACCACAGGGTAGAACCACGG
mmu-miR-146a-5p	TGAGAACTGAATTCCATGGGTT
mmu-miR-375-3p	TTTGTTCGTTCGGCTCGCGTGA
mmu-miR-199a-3p	ACAGTAGTCTGCACATTGGTTA
mmu-let-7d-3p	CTATACGACCTGCTGCCTTTCT
mmu-miR-21a-5p	TAGCTTATCAGACTGATGTTGA
mmu-miR-23a-3p	ATCACATTGCCAGGGATTTCC
mmu-miR-451a	AAACCGTTACCATTACTGAGTT
